# Long-acting bronchodilators improve exercise capacity in COPD patients: a systematic review and meta-analysis

**DOI:** 10.1186/s12931-018-0721-3

**Published:** 2018-01-24

**Authors:** Fabiano Di Marco, Giovanni Sotgiu, Pierachille Santus, Denis E. O’Donnell, Kai-Michael Beeh, Simone Dore, Maria Adelaide Roggi, Lisa Giuliani, Francesco Blasi, Stefano Centanni

**Affiliations:** 1 Respiratory Unit, Ospedale San Paolo, Department of Health Science, Università degli Studi di Milano, Via A. di Rudinì, 8-20142 Milan, Italy; 2 Clinical Epidemiology and Medical Statistics Unit, Department of Biomedical Sciences, University of Sassari, Sassari, Italy; 3 Department of Biomedical And Clinical Sciences (DIBIC), University of Milan, Milan, Italy; 4 Respiratory Unit, “Luigi Sacco” University Hospital; ASST Fatebenefratelli-Sacco, Milan, Italy; 5 Division of Respiratory and Critical Care Medicine, Respiratory Investigation Unit, Queen’s University and Kingston General Hospital, Kingston, ON Canada; 6 Insaf Respiratory Research Institute, Wiesbaden, Germany; 7 Cardiothoracic and Vascular Department, University of Pisa, Pisa, Italy; 8 Department of Pathophysiology and Transplantation, University of Milan, Milan, Italy; 9 Internal Medicine Department, Respiratory Unit and Cystic Fibrosis Adult Center, Fondazione IRCCS Ca’ Granda Ospedale Maggiore Policlinic, Milan, Italy

**Keywords:** COPD, Bronchodilator, Exercise

## Abstract

**Background:**

We carried out a systematic review and meta-analysis with the aim to evaluate the efficacy of long-acting bronchodilators on exercise capacity in COPD patients.

**Methods:**

The endpoints were the efficacy of long-acting bronchodilators (altogether, and by single classes) vs. placebo in modifying endurance time (ET), inspiratory capacity (IC) and dyspnea during exercise, taking into consideration the outcomes according to different patients’ inclusion criteria and exercise methodology.

**Results:**

Twenty-two studies were deemed eligible for analysis. Weighted mean increase in ET resulted of 67 s (95% CI ranges from 55 to 79). For isotime IC and dyspnea during exercise, weighted improvements were 195 ml (162–229), and − 0.41 units (− 0.56 to − 0.27), respectively. The increase in trough IC was 157 ml (138–175). We found a trend in favour of LAMA compared to LABA in terms of ET. In the 11 studies which reported a value of functional residual capacity > 120% as inclusion criterion, weighted mean increase in endurance time was 94 s (65 to 123); however we did not find any significant correlation between ET and mean trough IC (P: 0.593). The improvement of ET in the 5 studies using walking as exercise methodology resulted of 58 s (− 4 to 121).

**Conclusions:**

Long-acting bronchodilators improve exercise capacity in COPD. The main effect of long-acting bronchodilators seems to be a decrease of basal IC rather than a modification of dynamic hyperinflation during exercise. The efficacy in terms of endurance time seems higher in studies which enrolled patients with hyperinflation, with a similar efficacy on walking or cycling.

## Background

In patients with chronic obstructive pulmonary disease (COPD) exercise limitation is mainly due to dynamic hyperinflation [1], even if the contribution of other factors, such as an imbalance between respiratory and locomotor muscles for limited energy supply [2], limb muscle dysfunction [3], and co-morbidities (e.g., left ventricle diastolic dysfunction) [4] can play a significant role. Reduced daily activity has been well documented in COPD patients, resulting from both respiratory and non-respiratory clinical conditions associated with the disease [5]. An evaluation performed by multisensory armband confirmed that daily physical activity is mainly associated with dynamic hyperinflation, regardless of COPD severity [6]. It was clearly proved that exercise capacity and daily activity are closely associated with life expectancy [7]. Thus, improving physical activity represents the best approach to address both pulmonary and systemic manifestations of the disease [8].

Endurance time during high-intensity constant-load ergometer exercise protocols (i.e. 75–80% peak work-rate) is currently used to assess the effects of pharmacological and non-pharmacological interventions, as it has proven to be more sensitive than other procedures [9]. Importantly, endurance test is also listed as a suitable outcome for pivotal trials with pharmacological interventions by regulatory bodies, e.g. the Food and Drug Administration (FDA) and the European Medicines Agency (EMA), although no label claim for improvements in exercise capacity has been granted this far [10, 11]. Moreover, this approach allows an evaluation of symptoms intensity (e.g., dyspnea and leg effort), or physiological variables (e.g., inspiratory capacity, IC, assessment of dynamic hyperinflation) at a standardized time (isotime), which has been proved very useful in identifying the underlying physiological mechanisms responsible for modifications in exercise tolerance induced by a particular intervention [12].

In COPD exercise capacity can be improved by rehabilitative interventions [13], as well as interventions aimed at unloading the respiratory system, such as breathing heliox (i.e.) [14], oxygen therapy [15], or noninvasive ventilatory support [16], and recently high flow nasal cannula [17]. However, the first-line treatment for all COPD patients, after smoking cessation, remains pharmacological, mainly with inhaled medication. Long-acting bronchodilators represent the cornerstone of COPD pharmacological therapy [18, 19]. Long-acting muscarinic antagonists (LAMA), β_2_-agonists (LABA), alone or in combination with inhaled steroids (ICS), and the recent LABA/LAMA fixed-dose combinations (FDCs), have been evaluated to assess their efficacy on exercise capacity [8]. Despite the availability of studies on LABA, LAMA, and ICS/LABA or LABA/LAMA FDCs on exercise capacity, evidence for the efficacy of bronchodilators in enhancing the exercise capacity of patients with COPD is sometimes contradictory [20], with, for instance, a recent trial which failed to demonstrate the superiority of a LABA/LAMA FDC vs. placebo [21]; moreover, current literature is characterized by a large heterogeneity of studies, due to differences in terms of inclusion criteria (unselected patients vs. COPD patients with evidence of hyperinflation), and/or exercise methodology (cycling vs. walking, cycle or treadmill ergometer vs. shuttle walking test).

On this basis, we carried out a systematic review and meta-analysis on the available clinical evidence to evaluate the efficacy of long-acting bronchodilators (altogether, and by single classes) on exercise capacity, dynamic hyperinflation, and dyspnea during exercise using high-intensity constant-load exercise test both in unselected patients and in patients with demonstrated lung hyperinflation at rest, and in studies with different exercise methodologies (walking vs. cycling).

## Methods

This systematic review and meta-analysis was conducted according to the guidelines of the Preferred Reporting Items for Systematic Reviews and Meta-Analysis Protocols (PRISMA-P) statement [22].

### Search strategy

We selected randomized controlled studies (RCTs) focused on the efficacy of long-acting bronchodilators (i.e.: LABA, LAMA, LABA/ICS, and LABA/LAMA FDCs) on exercise capacity in COPD patients based on endurance time with high-intensity constant-load performed on a cycle or treadmill and whose duration was longer than one week. We searched in PubMed and EMBASE through September 2017. The following keywords and their related MeSH (Medical Subjects Heading) terms were used: “chronic obstructive pulmonary disease”, “COPD”, “bronchodilator”, “long-acting bronchodilator”, “LABAs”, “LAMAs”, “cardiopulmonary exercise test”, “endurance time”, and “exercise”. Only publications in English language were considered.

### Studies selection and data extraction and study quality assessment

We included RCTs focused on COPD patients (any level of severity) which assessed as a primary or secondary outcome the efficacy of long-acting bronchodilators on exercise capacity evaluated by “endurance time” using high-intensity constant-load ergometer exercise test, or shuttle walking test, providing the results of active drugs and placebo and not only between-arms difference. The shuttle walking test uses an audio signal from a tape cassette to direct the walking pace of the patient back and forth on a 10-m course. Test ends when the patient cannot reach the turnaround point within the required time [12]. We included studies which used cycling or walking. In both cases maximal exercise capacity was measured using incremental symptom limited exercise test. Subsequently, constant-load (cycling or walking at 75–90% of maximal work load) cycling ergometer or shuttle walking test (ESWT) were used to compare the efficacy of active drugs with placebo. The following exclusion criteria were chosen: 1) manuscripts focused on short-acting bronchodilators, or not including placebo; 2) exercise capacity assessment based on a protocol different from high-intensity constant load exercise test; 3) epidemiological observational study design or experimental design other than RCT; 4) manuscripts not written in English language; 5) data expressed only as difference vs. placebo and not as single arms (active drugs and placebo). Two independent authors (MAR and FDM) firstly reviewed all titles/abstracts to identify potentially relevant articles. Then, study selection, based on a full-text review, was performed according to the predefined inclusion/exclusion criteria and disagreements were resolved by consensus. The same authors reviewed eligible studies using the CONSORT quality standard, judged the studies quality by Jadad scale [23, 24], and the risk of bias by a domain-based evaluation, which included the following domains: sequence generation, allocation concealment, blinding of participants and personnel, blinding of outcome assessment, attrition, selective reporting bias and other sources of bias. Explicit judgments were made about the overall risk of bias according to the Cochrane guidance [25].

### Endpoints

The primary endpoint was to assess the efficacy of all long-acting bronchodilators (i.e.: LABA, LAMA, LABA/ICS FDCs, and LABA/LAMA FDCs) vs. placebo on endurance time. The secondary endpoints were to investigate the role of the above-mentioned drugs on IC and dyspnea, as well as the comparison between drug classes in terms of endurance time, IC, and dyspnea.

### Statistical analysis

Forest plots were created to graphically assess both the variability of the sample estimates and the weight of sample sizes in the computation of estimates (weighted means). A random-effects meta-analysis was carried out to account for the presumed high between-study variability. Inconsistency among studies was assessed by the I^2^ statistic to underscore the effect of true variability on the overall variation. Since the inclusion criteria of the studies in this field sometimes include the presence of hyperinflation (i.e. FRC > 120%) a subgroup analysis was carried out; furthermore, sub-analyses of the endurance time were performed according to the exercise methodology (i.e. cycling vs. walking). Funnel plots and their related Egger’s tests were performed to visually assess the risk of bias, particularly publication and small sample bias. Correlation between endurance time and change in relevant physiological variables was undertaken. The statistical software used were Stata13.0 (StataCorp, College Station, TX, USA) and StatsDirect 2.8.0, version 1.4 (StatsDirect Ltd., Altrincham, UK).

## Results

**Fig. 1:**
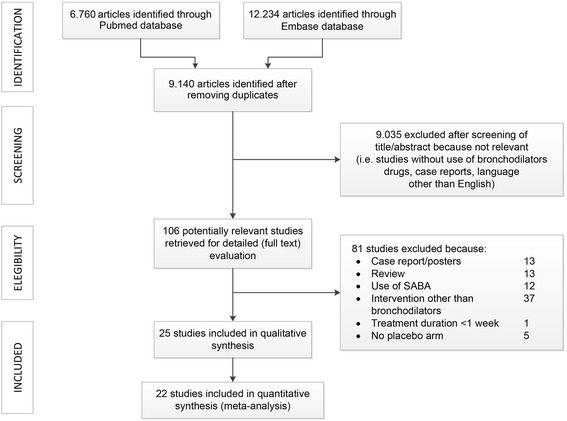
PRISMA (Preferred Reporting Items for Systematic Reviews and Meta-Analyses) flow diagram

Out of 88 potentially relevant studies, 22 (25%) were deemed eligible for a qualitative and quantitative analysis (Fig. 1); their characteristics are summarized in Table 1 [21, 26–44]. The efficacy of LABA, LAMA, LABA/LAMA FDCs, and ICS/LABA FDCs as main treatment was assessed in 6 (27%), 10 (45%), 3 (14%), and 3 (14%) studies, respectively, with 5 (23%) studies using another active drug as control of the experimental arm (Table 1). The study design was cross-over in the majority of the cases (15 studies, 68%). Endurance time was the most frequently adopted primary outcome, followed by pulmonary function; mean duration of treatment was 8.9 ± 15.9 weeks. The methodology used for exercise and the intensity of the constant workload are reported in Table 1; in most cases cycle ergometer exercise testing was performed at 75–90% of maximal work load. The BORG scale was used to evaluate dyspnea in all studies. Characteristics of the enrolled patients are reported in Table 2. Total sample size included 2898 patients; 65.4 ± 9.1% of which were males, with a mean age (mean SD) of 62.9 (7.7) years, BMI of 26.7 (4.4) Kg/m^2^, basal FEV_1_ of 50.3 (11.5)% of predicted value, and a basal inspiratory capacity of 78.4 (19.7) % of predicted value. Weighted mean increase of trough FEV_1_ and trough IC following the exposure to all long-acting bronchodilators (end of the study) selected in out meta-analysis resulted of 144 ml (95% CI ranges from 126 to 162; I^2^: 73.4% treatment arms: 40), and of 157 ml (95% CI ranges from 138 to 175; I^2^: 34.8% treatment arms: 30), respectively.

**Table 1:** Characteristics of selected studies

Author, year	Country	Study design	Main treatment	Other active drugs	Duration (weeks)	Primary outcome	Secondary outcomes	Exercise methodology and intensity
*Man WDC, 2004*	UK	Crossover	Salmeterol 50 μg bid	–	2	Transdiaphragmatic pressure	Endurance time. Pulmonary function, dyspnoea	Treadmill, 80% W
*O’Donnell DE, 2004*	Canada	Crossover	Salmeterol 50 μg bid	–	2	Pulmonary function and dyspnoea	Endurance time	Bike, 75% W
*Neder JA, 2007*	Brazil, UK	Crossover	Formoterol 12 μg bid	–	2	Endurance time	Pulmonary function, dyspnoea	Bike 80%, W
*Beeh KM, 2011*	Germany, UK	Crossover	Indacaterol 300 μg od	–	2	Pulmonary function	Endurance time, dyspnoea	Bike 80%, W
*O’Donnell DE, 2011*	Canada, Belgium, Spain, USA, UK	Crossover	Indacaterol 300 μg od	–	3	Endurance time	Pulmonary function, dyspnoea	Bike 75%, W
*Maltais F, 2016*	Germany	Crossover	Olodaterol 5, 10 μg	–	6	Endurance time	Pulmonary function, dyspnoea	Bike 75%, W
*O’Donnell DE, 2004*	Canada, Germany, USA	Parallel	Tiotropium 18 μg od	–	6	Endurance time	Pulmonary function, dyspnoea	Bike 75%, W
*Casaburi R, 2005*	USA	Parallel	Tiotropium 18 μg od	–	25	Endurance time	Pulmonary function, dyspnoea	Treadmill, 80% W
*Maltais F, 2005*	Canada, USA	Parallel	Tiotropium 18 μg od	–	6	Endurance time	Pulmonary function, dyspnoea	Bike, 75% W
*Travers J, 2007*	Canada, USA	Crossover	Tiotropium 18 μg od	–	1	Cardiopulmonary function	Endurance time	Bike, 75% W
*Maltais F, 2011*	Canada, USA, Spain	Parallel	Aclidinium 200 μg od	–	6	Endurance time	Pulmonary function, dyspnoea	Bike, 75% W
*Beeh KM, 2012*	Germany, UK, Switzerland	Crossover	Glycopyrronium 50 μg od	–	3	Endurance time	Pulmonary function, dyspnoea	Bike, 80% W
*Cooper CB, 2013*	USA, UK, Brazil	Parallel	Tiotropium 18 μg od	–	96	Endurance time	Pulmonary function, dyspnoea	Treadmill 90% W^a^
*Beeh KM, 2014*	Germany	Crossover	Aclidinium 400 μg bid	–	3	Endurance time	Pulmonary function, dyspnoea	Bike, 75% W
*Casaburi R, 2014*	USA, Canada	Crossover	Tiotropium 18 μg od	–	6	IC isotime	Endurance time, Borg isotime	Treadmill, 80% W^a^
*Bedard M-E, 2012*	Canada	Parallel	Tiotropium 18 μg od	–	3	Endurance time	Pulmonary function, dyspnoea	ESWT, 80% VO2
*Beeh KM, 2014*	Germany, UK, USA	Crossover	Indacaterol/Glycopyrronium 110/50 μg od	Tiotropium 18 μg od	3	Endurance time	Pulmonary function, dyspnoea	Bike, 75% W
*Maltais F, 2014*	Germany	Crossover	Umeclidinium/vilanterol 125/25, 62,5/25 μg	Vilanterol 25 μg, Umeclidinium 62,5, 125 μg	12	Endurance time, trough FEV_1_	Pulmonary function	ESWT, 80–90% speed
*O’Donnell, 2017*		Crossover	Tiotropium/Olodaterol, 5/2,5, 5/5 μg	Tiotropium 5 μg, Olodaterol 5 μg	6	Endurance time, inspiratory capacity	Pulmonary function, dyspnea	Bike, 75% W
*O’Donnell DE, 2006*	Canada, USA	Parallel	Salmeterol 50 μg/Fluticasone 250 μg bid	Salmeterol 50 μg bid	8	Pulmonary function and dyspnoea	Endurance time	Bike, 75% W
*Worth H, 2010*	Germany, Sweden	Crossover	Budesonide/formoterol 320/9 μg bid	Formoterol 9 μg bid	1	Endurance time	Pulmonary function, dyspnea	Bike, 75% W
*Guenette JA, 2013*	Canada	Crossover	Fluticasone 250/Salmeterol 50 μg bid	–	6	Endurance time	Pulmonary function, dyspnoea	Bike, 85% W

*Od* Once daily, *Bid* Twice daily, *ESWT* Endurance shuttle walking test, *W* Work load. ^a^Work rate was adjusted to obtain an exercise duration between a specified time interval

**Table 2:** Characteristics of patients enrolled in studies selected for the final analysis

Author, year		Main treatment	Inclusion criteria	Numbers of individuals randomized	Number available for the final analysis	Male, %	Age, yrs	BMI, Kg/m^2^	Basal FEV_1_, %
*Man WDC, 2004*		Salmeterol 50 μg bid	FEV_1_ change post bd < 10% and 200 ml	20	16	63	68 (7.6)	–	31.1 (3.9)
*O’Donnell DE, 2004*		Salmeterol 50 μg bid	FEV_1_ ≤ 70%, FRC ≥ 120%, BDI ≤ 6	23	23	65	64 (2.0)	26.1 (0.8)	42 (−)
*Neder JA, 2007*		Formoterol 12 μg bid	FEV_1_/FVC ≤ 60%, FEV_1_ < 60% and change after bd < 12%	21	18	67	42–75 (range)	24.8 (5.1)	38.8 (11.7)
*Beeh KM, 2011*		Indacaterol 300 μg od	40–80 yrs., 80% ≥ FEV_1_ ≥ 40%, FRC ≥ 120%	27	24	67	61.3 (7.2)	25.6 (3.4)	51.5 (11.4)
*O’Donnell DE, 2011*		Indacaterol 300 μg od	≥40 yrs., 80% ≥ FEV_1_ ≥ 30%	90	74	70	62.8 (8.2)	27.5 (4.1)	61 (12.4)
*Maltais F, 2016*	Study 1222.37	Olodaterol 5, 10 μg	40–75 yrs., FEV_1_ < 80%	151	140	77	60.6 (7.7)	–	48.5 (14.5)
	Study 1222.38			157	141	74	60.6 (7.7)	–	51.6 (14.2)
*O’Donnell DE, 2004*		Tiotropium 18 μg od	40–70 yrs., FEV_1_ ≤ 65%, FRC ≥ 120%	198	187	74	60.5 (−)	26.5 (4.8)	44 (13.0)
*Casaburi R, 2005*		Tiotropium 18 μg od	≥40 yrs., FEV_1_ ≤ 60%	108	91	57	66.6 (7.9)	25.9 (5.2)	34.4 (12.4)
*Maltais F, 2005*		Tiotropium 18 μg od	40–75 yrs., FEV_1_ ≤ 65%, FRC ≥ 120%	261	241	72	62.5 (7.4)	–	43.1 (12.7)
*Travers J, 2007*		Tiotropium 18 μg od	FEV_1_ ≤ 65%, FRC ≥ 120%, BDI ≤ 6	–	18	72	60 (9.0)	26.8 (5.4)	40 (−)
*Maltais F, 2011*		Aclidinium 200 μg od	≥40 yrs., 80% ≥ FEV_1_ ≥ 30%, FRC ≥ 120%, BDI ≤ 7	181	159	52	64.8 (−)	26.4 (−)	50 (−)
*Beeh KM, 2012*		Glycopyrronium 50 μg od	≥40 yrs., 80% ≥ FEV_1_ ≥ 40%	108	95	58	60.5 (8.6)	26.6 (4.0)	57.1 (8.5)
*Cooper CB, 2013*		Tiotropium 18 μg od	≥40 yrs., FEV_1_ ≤ 65%, mMRC≥2	519	464	77	65 (−)	26.4 (−)	44 (12.0)
*Beeh KM, 2014*		Aclidinium 400 μg bid	≥40 yrs., 80% ≥ FEV_1_ ≥ 30%, FRC ≥ 120%	112	106	68	60.3 (8.1)	–	56.7 (11.6)
*Casaburi R, 2014*		Tiotropium 18 μg od	≥40 yrs., FEV_1_ ≥ 50%, 35 ≥ BMI ≥ 18 Kg/m^2^, BDI ≤ 9	126	111	52	61.2 (8.8)	27.8 (3.9)	70 (17.0)
*Bedard M-E, 2012*		Tiotropium 18 μg od	≥50 yrs., FEV_1_ < 70%	36	34	68	65 (7)	28 (4)	54 (12)
*Beeh KM, 2014*		Indacaterol/Glycopyrronium 110/50 μg od	≥40 yrs., 70% ≥ FEV_1_ ≥ 40%	85	73	63	62.1 (8.1)	–	56 (8.9)
*Maltais F, 2014*	Study 417	Umeclidinium/vilanterol 125/25, 62,5/25 μg	≥40 yrs., 70% ≥ FEV_1_ ≥ 35%, FRC ≥ 120%, mMRC≥2	349	348	56	61.6 (8.3)	–	51.3 (9.7)
	Study 418			308	307	55	62.6 (7.9)	–	51.3 (10.0)
*O’Donnell, 2017*	Mor 1	Olodaterol/Tiotropium 2,5/5, 5/5 μg	40–75 yrs., 80% ≥ FEV_1_ ≥ 30%	295	227	72	62.2 (7.5)	27.3 (5.3)	52.6 (13.9)
	Mor 2			291	224	70	61.2 (7.9)	26.7 (4.6)	52.0 (13.4)
*O’Donnell DE, 2006*		Salmeterol 50 μg/Fluticasone 250 μg bid	≥40 yrs., FEV_1_ < 70%, FRC ≥ 120%, BDI < 7, ≥20 W at CPET	123	117	70	64 (−)	25.9 (−)	41 (−)
*Worth H, 2010*		Budesonide/formoterol 320/9 μg bid	≥exacerbation last 1 yr., FEV_1_ ≤ 50%, FRC > 120%	111	91	76	63.7 (−)	25.7 (−)	37 (8.4)
*Guenette JA, 2013*		Fluticasone 250/Salmeterol 50 μg bid	≥40 yrs., FEV_1_ > 60%	18	15	40	64 (10.0)	29.5 (6.4)	86 (15.0)

*Od* Once daily, *bid* Twice daily, *bd* Bronchodilation, *Mor* MORACTO study

The median Jadad score for the RCTs included in our analysis was 4 (range 4–5); a detailed assessment of the risk of bias is described in the supplement materials using the Cochrane collaboration tool for assessing risk of bias. The risk of bias was deemed low for the majority of the items in the selected studies. No relevant asymmetries were found in several funnel plots related to the main clinical variables.

### Efficacy of long-acting bronchodilators on ET, inspiratory capacity, and dyspnea during exercise

**Fig. 2:**
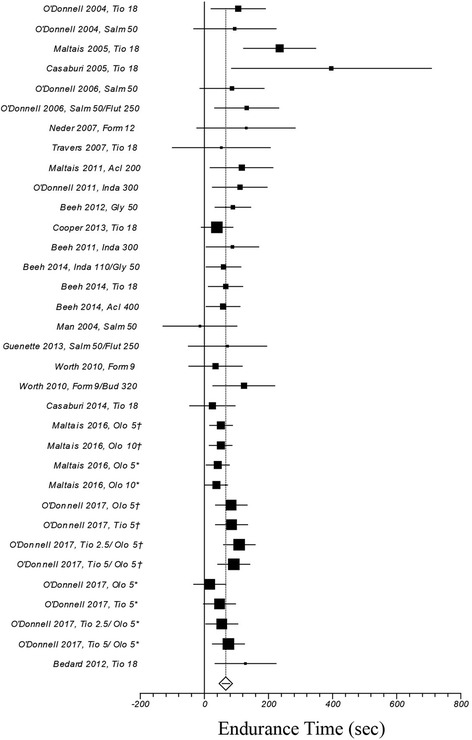
Efficacy of long-acting bronchodilators on endurance time. I^2^ 21.1% (95% CI 0–48.8%). Error bars represent 95% confidence intervals. †: study n. 1222.37; *: study n. 1222.38

**Fig. 3:**
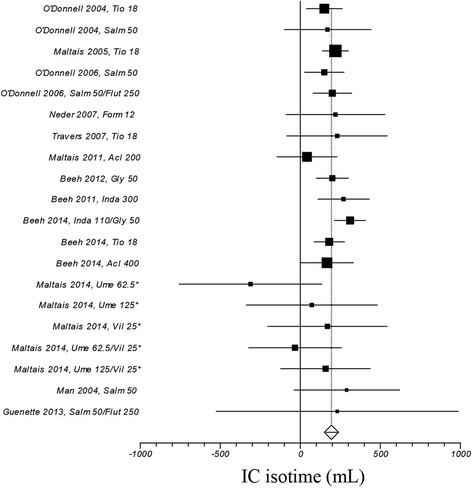
Efficacy of long-acting bronchodilators on isotime inspiratory capacity. I^2^ 1.2% (95% CI 0–42.8%). Error bars represent 95% confidence intervals. *: study n. 417

**Fig. 4:**
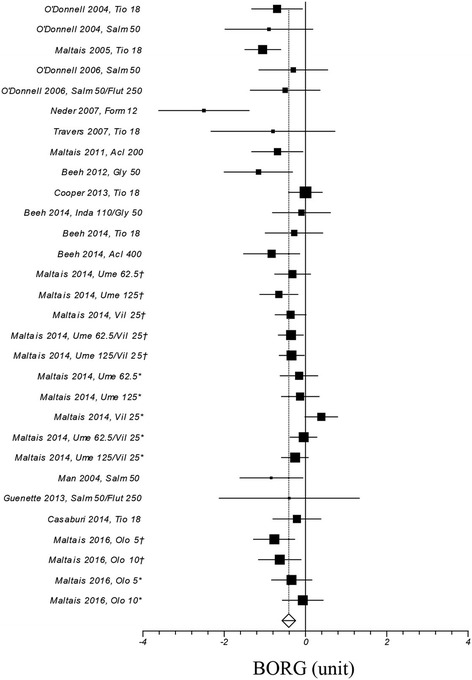
Efficacy of long-acting bronchodilators on dyspnea. I^2^ 55.1% (95% CI 26.8–69.4%). Error bars represent 95% confidence intervals. For Ume/Vil †: study n. 1222.37; *: study n. 1222.38. For Olo †: study n. 418; *: study n. 417

Figure 2 illustrates a weighted mean increase in endurance time following the exposure to long-acting bronchodilators of 67 s (95% CI ranges from 55 to 79; I^2^: 22.1%, computed on 34 treatment arms). The role played by long-acting bronchodilators on isotime IC and dyspnea is summarized in Figs. 3, and 4, respectively: weighted means were 195 ml (95% CI ranges from 162 to 229; I^2^: 1.2%; treatment arms: 20), and − 0.41 units (95% CI ranges from − 0.56 to − 0.27; I^2^: 55.1%; treatment arms: 30), respectively.

### Efficacy of different classes of long-acting bronchodilators

**Fig. 5:**
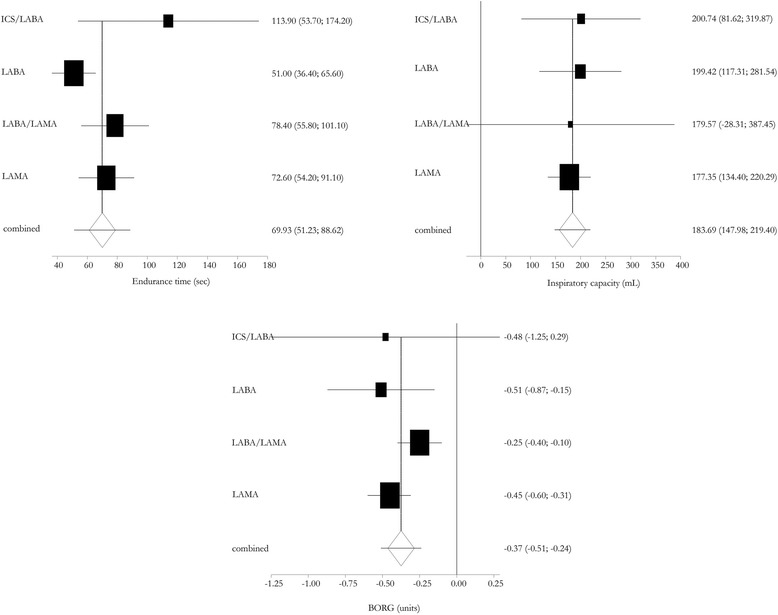
Efficacy of different classes of long-acting bronchodilators tested at the approved dose for COPD treatment. Error bars represent 95% confidence intervals

Figure 5 shows the efficacy of different classes of long-acting bronchodilators at approved doses for COPD treatment in terms of endurance time, isotime IC, and dyspnea. This analysis did not show any significant differences among the investigated categories of long-acting bronchodilators; however, for endurance time the efficacy of LABA seems lower than for other classes.

### Efficacy of long-acting bronchodilators in patients with hyperinflation and using different exercise methodologies

To better understand the role of hyperinflation at rest we performed a prespecified subgroup analysis focused on the 11 studies which reported a value of FRC > 120% as inclusion criterion (Table 2). In this subgroup of studies, mean change of trough FEV_1_ following the exposure to all long-acting bronchodilators (end of the study) resulted of 153 ml (95% CI ranges from 123 to 183; I^2^: 73.8% treatment arms: 19), and mean change of trough IC resulted of 154 ml (95% CI ranges from 121 to 187; I^2^: 50.4% treatment arms: 18). Weighted mean change of endurance time, inspiratory capacity, and dyspnea following the exposure to long-acting bronchodilators resulted of 94 s (95% CI ranges from 65 to 123; I^2^: 10.4%, computed on 11 treatment arms), 174 ml (95% CI ranges from 131 to 216; I^2^: 0%; treatment arms: 14), and − 0.37 units (95% CI ranges from − 0.54 to − 0.21; I^2^: 53.2%; treatment arms: 18), respectively (Additional file 1: Figure S2A). In the subgroup of the 11 studies which did not require an increase of FRC as inclusion criterion weighted mean change of endurance time, inspiratory capacity and dyspnea following the exposure to long-acting bronchodilators resulted of 61 s (95% CI ranges from 49 to 73; I^2^: 17.2%, computed on 23 treatment arms), 231 ml (95% CI ranges from 178 to 285; I^2^: 0%; treatment arms: 6), and − 0.51 units (95% CI ranges from − 0.80 to − 0.21; I^2^: 60.2%; treatment arms: 12), respectively (Additional file 1: Figure S2B). We did not find any significant correlations between ET and mean trough FEV_1_ (rho: 0.38, P: 0.063), or between ET and trough IC (rho: 0.14, P: 0.593).

In the subgroup of the 5 studies which used walking as exercise methodology, weighted mean change of endurance time following the exposure to long-acting bronchodilators resulted of 58 s (95% CI ranges from − 4 to 121; I^2^: 56.2%, computed on 5 treatment arms), compared to 68 s (95% CI ranges from 56 to 79; I^2^: 13.3%, computed on 29 treatment arms) in the studies which used a cycle ergometry (Additional file 1: Figure S3).

## Discussion

The main findings of this systematic review and meta-analysis are: 1) long-acting bronchodilators are effective in improving exercise capacity in COPD patients, with an average increase of about 60 s; 2) this effect is associated with a reduction of dyspnea during exercise; 3) the increase in isotime IC is similar to the change of trough IC, and this is the reason why the effect seems to be a decrease of basal inspiratory capacity rather than a modification of dynamic hyperinflation during exercise; 4) the use of LABA and LAMA is associated with a similar improvement of exercise tolerance, dynamic hyperinflation, and dyspnea; however, it is possible to identify a trend in favour of LAMA in terms of ET; 5) the efficacy of long-acting bronchodilators on ET were higher in the studies which required an increase of FRC as inclusion criterion; however we failed to demonstrate a correlation between ET and trough IC; 6) the efficacy of long-acting bronchodilators is similar when walking or cycling are used as exercise methods.

Maintenance therapy with long-acting bronchodilators has been demonstrated to significantly reduce operational lung volumes during exercise in symptomatic patients with COPD, even if this beneficial effect does not always have an impact on exercise duration. Casaburi R et al. found a significant effect of tiotropium on exercise duration in COPD patients with moderate (i.e. FEV_1_ < 80%), but not mild (i.e. FEV_1_ > 80%) disease [40]. Our meta-analysis found that, on average, there is a concordance between the increase of inspiratory capacity, the improvement of exercise tolerance, and the reduction of dyspnea in patients with moderate to severe COPD. Unfortunately, we are not able to extend these findings to patients with mild disease, since most of the studies we included in our analysis had enrolled patients with FEV_1_ < 70% predicted value (Table 2), and also in case of inclusion criteria permissive for milder disease, results for patients with FEV_1_ > 80% were not available. As previously discussed, we found a significantly higher improvement in ET, of about 50%, in the studies which required an increase of FRC as inclusion criterion; this result could demonstrate that long-acting bronchodilators are more effective in terms of exercise capacity in COPD patients with lung hyperinflation. Thus, even if we found that the improvement of operational lung volume is correlated with the increase of ET after bronchodilation, we failed to demonstrate a correlation between trough IC and ET per se, result which could suggest that, even if hyperinflation is a crucial aspect for exercise limitation in COPD, the absolute value of ET depends on many factors.

We did not find any significant difference in terms of endurance time, dynamic hyperinflation, and symptoms during exercise between LABA and LAMA, the two classes of long-acting bronchodilators most studied in these fields; however, a trend in favor of LAMA was identifiable at least in terms of endurance time. It’s to notice that the present study does not allow to explain why LAMA could be superior of LABA in terms of exercise capacity. Beta-2 agonists and antimuscarinic agents could theoretically exert a different efficacy on exercise capacity due to differences in the distribution of receptors, both in and outside the lung, and in mechanism of action. Indeed, beta-2 adrenergic receptors are present in high density not only in airway smooth muscle cells, but also in vascular endothelium, ciliated cells, circulating inflammatory cells, such as eosinophils, and sub-mucosal glands. On the other hand, muscarinic receptors are present on bronchial smooth muscle cells, parasympathetic and sympathetic nerves, ganglia cells, mucus secreting cells, and inflammatory cells. ICS/LABA FDCs have proved to be more effective than LABA alone in terms of function, quality of life, symptoms and prevention of exacerbations [45]. Therefore, even if the efficacy on FEV_1_ cannot be directly translated to lung deflation (a crucial determinant of exercise capacity on COPD), there is a rationale to expect a better efficacy of ICS/LABA FDCs rather than LABA alone on exercise capacity. The same, and probably with major emphasis, has to be expected for LABA/LAMA combinations, which confirmed to be more effective in terms of function and symptoms than not only LABA or LAMA alone, but also ISC/LABA FDCs [46, 47]. Even if some of the studies currently available failed to demonstrate a superiority of LABA/LAMA compared with mono-components [21, 41], in a recent meta-analysis LABA/LAMA FDCs were found to be more effective than LABA or LAMA alone in terms of ET [48]. There is limited information on minimum clinically significant differences for endurance time after an intervention [49]. In COPD, a 105 s change from baseline using cycle ergometry related well with positive patient-reported outcomes after pulmonary rehabilitation [50, 51]. However, data from bronchodilator studies suggest that improvements in lung function that seem to be clinically important are often associated with increases in endurance time of 60 s [49]. For dyspnea (isotime Borg) and IC at isotime, the minimal clinically important difference (MCID) were suggested to be 1 unit, and 200 ml, respectively [49]. Our meta-analysis found a weighted mean change, following the exposure to long-acting bronchodilators, of endurance time, dyspnea, and IC at isotime of 67 s, − 0.41 units, and 195 ml, respectively. These improvements we found are higher than the MCID for endurance time, probably the most important outcome, borderline clinically significant for dynamic hyperinflation during exercise, but far from the MCID for dyspnea. The average improvement we found is similar to that obtained by oxygen in mildly or nonhypoxaemic COPD patients who are dyspnoeic at rest [52]. It is noteworthy that, as pointed out by Puente-Maestu et al. in a recent ERS statement, nonpharmacological interventions, such as rehabilitation, Heliox, oxygen in hypoxaemic patients and noninvasive ventilation, have demonstrated significantly higher endurance time increases [49]. However, pharmacological and nonpharmacological treatments of COPD are not mutually exclusive, since the former have to be considered in all cases, and their combination has shown to be more convenient that a single approach [33]. Moreover, the evaluation of variables at isotime is sometimes problematic since they are often extrapolated or interpolated.

Finally, we found a similar efficacy of long-acting bronchodilators when walking or cycling are used as exercise methodologies. The main reason why the sub-analysis of the 5 studies which used walking as exercise methodology failed to demonstrate a statistically significant efficacy vs. placebo is probably due to the low power of the analysis, with an average difference similar to that of the studies which used cycle ergometer (58 vs. 68 s). This result, important since “walking” is evidently the most common real-life exercise of COPD patients, confirms a previous study, which demonstrated a similar efficacy of formoterol on endurance time evaluated by walking and cycling [53].

A number of potential limitations of the study deserves discussion. Firstly, the studies we included in this meta-analysis present different inclusion criteria for COPD patients who, in most but not all studies, were requested to present a significant hyperinflation (e.g. FRC > 120% of predicted values); this could limit the external validity of our analysis, since the main indication of long-acting bronchodilators is so far COPD per se (i.e. FEV_1_/FVC < lower limit of normal) without the need of hyperinflation. However, a specific analysis including the studies which requested an increase of FRC as inclusion criterion was performed, with evidence of a higher efficacy of long-acting bronchodilators when compared with the studies which evaluated unselected COPD patients. Secondly, the duration of the studies we included in our analysis was very heterogeneous, ranging from one to 96 weeks, with an average duration of the treatment of 9.6 weeks. Probably depending on the onset of action, some drugs demonstrated to be able to improve endurance time immediately after the first inhalation [29]; however, due to the chronic nature of the disease and the consequent long treatment course, this aspect is not so clinically relevant, and a significant effect expected in “some” weeks is the message which emerges from our analysis. Then, we decided to include ICS/LABA combination in the analysis, since this treatment is very common in clinical practice. The addition of ICS to LABA can be a confounder for the interpretation of the effect of long-acting bronchodilators on exercise capacity. However, the subgroups analysis according to treatment classes (LABA; LAMA, ICS/LABA, and LABA/LAMA) allows to evaluate the effects of bronchodilators alone, confirming the efficacy of these treatment without ICS. Finally, the changes we found of isotime IC are close to those of trough IC, suggesting that the main effect of long-acting bronchodilators is an increase in basal inspiratory capacity rather than a modification of dynamic hyperinflation during exercise (i.e. the slope of change of IC).

## Conclusion

Long-acting bronchodilators improve exercise capacity in COPD patients, with an average change higher than the minimum clinically significant difference. While long-acting bronchodilators consistently improve the capacity to tolerate exercise by improving lung mechanics, the challenge remains to convert this advantage into increased habitual physical activity. An additional behavioral intervention will likely be needed to achieve this in the hope of improving long-term survival [8].

## Additional file

Additional file 1: Figure S1. Funnel plots for ET, isotime IC and isotime dyspnea. **Figure S2A** Forest and Funnel plots of ET, isotime IC and isotime dyspnea in the 11 studies which included only COPD patients with functional residual capacity (FRC) > 120%. **Figure S2B** Forest and Funnel plots of ET, isotime IC and isotime dyspnea in the 11 studies which did not require an increase of functional residual capacity (FRC) as inclusion criterion (i.e. unselected COPD patients). **Figure S3** Forest and Funnel plots of ET of the 5 studies (A) which used walking, and 17 studies (B) which used cycling as exercise methodology to assess the efficacy of long-acting bronchodilators. **Table S1** Cochrane collaboration tool for assessing risk of bias. (DOCX 545 kb)

## Abbreviations

COPD: Chronic obstructive pulmonary disease
CPET: Cardiopulmonary exercise test
EMA: European Medicines Agency
ET: Endurance time
FDA: Food and Drug Administration
FDC: Fixed-dose combinations
FEV_1_: Forced expiratory volume in the first second
FRC: Functional residual capacity
IC: Inspiratory capacity
ICS: Inhaled steroids
LABA: β_2_-agonists
LAMA: Long-acting muscarinic antagonists
MeSH: Medical Subjects Heading
PRISMA: Preferred Reporting Items for Systematic Reviews and Meta-Analysis
RCT: Randomized controlled study

## References

[1] O'Donnell DE, Webb KA (2008). The major limitation to exercise performance in COPD is dynamic hyperinflation. J Appl Physiol (1985).

[2] Aliverti A, Macklem PT (2008). The major limitation to exercise performance in COPD is inadequate energy supply to the respiratory and locomotor muscles. J Appl Physiol (1985).

[3] Debigare R, Maltais F: The major limitation to exercise performance in COPD is lower limb muscle dysfunction**.** J Appl Physiol (1985) 2008, 105**:**751–753; discussion 755-757. 10.1152/japplphysiol.90336.2008a 18678623

[4] Lopez-Sanchez M, Munoz-Esquerre M, Huertas D, Gonzalez-Costello J, Ribas J, Manresa F, Dorca J, Santos S (2013). High prevalence of left ventricle diastolic dysfunction in severe COPD associated with a low exercise capacity: a cross-sectional study. PLoS One.

[5] Pitta F, Troosters T, Spruit MA, Probst VS, Decramer M, Gosselink R (2005). Characteristics of physical activities in daily life in chronic obstructive pulmonary disease. Am J Respir Crit Care Med.

[6] Garcia-Rio F, Lores V, Mediano O, Rojo B, Hernanz A, Lopez-Collazo E, Alvarez-Sala R (2009). Daily physical activity in patients with chronic obstructive pulmonary disease is mainly associated with dynamic hyperinflation. Am J Respir Crit Care Med.

[7] Waschki B, Kirsten A, Holz O, Muller KC, Meyer T, Watz H, Magnussen H (2011). Physical activity is the strongest predictor of all-cause mortality in patients with COPD: a prospective cohort study. Chest.

[8] Di Marco F, Santus P, Sotgiu G, Blasi F, Centanni S (2015). Does improving exercise capacity and daily activity represent the holistic perspective of a new COPD approach?. COPD.

[9] Oga T, Nishimura K, Tsukino M, Hajiro T, Ikeda A, Izumi T (2000). The effects of oxitropium bromide on exercise performance in patients with stable chronic obstructive pulmonary disease. A comparison of three different exercise tests. Am J Respir Crit Care Med.

[10] EMA/CHMP/700491/2012; http://www.ema.europa.eu/ema/.

[11] FDA Guidance for Industry: COPD dMwfg.

[12] ERST F, Palange P, Ward SA, Carlsen KH, Casaburi R, Gallagher CG, Gosselink R, O'Donnell DE, Puente-Maestu L, Schols AM (2007). Recommendations on the use of exercise testing in clinical practice. Eur Respir J.

[13] Troosters T, Casaburi R, Gosselink R, Decramer M (2005). Pulmonary rehabilitation in chronic obstructive pulmonary disease. Am J Respir Crit Care Med.

[14] Palange P, Valli G, Onorati P, Antonucci R, Paoletti P, Rosato A, Manfredi F, Serra P (2004). Effect of heliox on lung dynamic hyperinflation, dyspnea, and exercise endurance capacity in COPD patients. J Appl Physiol (1985).

[15] Bradley JM, Lasserson T, Elborn S, Macmahon J, O'Neill B (2007). A systematic review of randomized controlled trials examining the short-term benefit of ambulatory oxygen in COPD. Chest.

[16] Maltais F, Reissmann H, Gottfried SB (1995). Pressure support reduces inspiratory effort and dyspnea during exercise in chronic airflow obstruction. Am J Respir Crit Care Med.

[17] Cirio S, Piran M, Vitacca M, Piaggi G, Ceriana P, Prazzoli M, Paneroni M, Carlucci A (2016). Effects of heated and humidified high flow gases during high-intensity constant-load exercise on severe COPD patients with ventilatory limitation. Respir Med.

[18] Global Initiative for Chronic Obstructive Lung Disease. Global strategy for diagnosis, management and prevention of COPD. goldcopd.org.

[19] Bettoncelli G, Blasi F, Brusasco V, Centanni S, Corrado A, De Benedetto F, De Michele F, Di Maria GU, Donner CF, Falcone F (2014). The clinical and integrated management of COPD. Sarcoidosis Vasc Diffuse Lung Dis.

[20] Aguilaniu B (2010). Impact of bronchodilator therapy on exercise tolerance in COPD. Int J Chron Obstruct Pulmon Dis.

[21] Maltais F, Singh S, Donald AC, Crater G, Church A, Goh AH, Riley JH (2014). Effects of a combination of umeclidinium/vilanterol on exercise endurance in patients with chronic obstructive pulmonary disease: two randomized, double-blind clinical trials. Ther Adv Respir Dis.

[22] Moher D, Liberati A, Tetzlaff J, Altman DG, Group P (2009). Preferred reporting items for systematic reviews and meta-analyses: the PRISMA statement. PLoS Med.

[23] Jadad AR, Moore RA, Carroll D, Jenkinson C, Reynolds DJ, Gavaghan DJ, HJ MQ (1996). Assessing the quality of reports of randomized clinical trials: is blinding necessary?. Control Clin Trials.

[24] Schulz KF, Altman DG, Moher D, Group C (2010). CONSORT 2010 statement: updated guidelines for reporting parallel group randomised trials. J Clin Epidemiol.

[25] Higgins JP, Altman DG, Gotzsche PC, Juni P, Moher D, Oxman AD, Savovic J, Schulz KF, Weeks L, Sterne JA (2011). The Cochrane Collaboration's tool for assessing risk of bias in randomised trials. BMJ.

[26] Man WD, Mustfa N, Nikoletou D, Kaul S, Hart N, Rafferty GF, Donaldson N, Polkey MI, Moxham J (2004). Effect of salmeterol on respiratory muscle activity during exercise in poorly reversible COPD. Thorax.

[27] O'Donnell DE, Voduc N, Fitzpatrick M, Webb KA (2004). Effect of salmeterol on the ventilatory response to exercise in chronic obstructive pulmonary disease. Eur Respir J.

[28] Neder JA, Fuld JP, Overend T, Thirlwell J, Carter R, Stevenson R, Ward SA (2007). Effects of formoterol on exercise tolerance in severely disabled patients with COPD. Respir Med.

[29] Beeh KM, Wagner F, Khindri S, Drollmann AF (2011). Effect of indacaterol on dynamic lung hyperinflation and breathlessness in hyperinflated patients with COPD. Copd.

[30] O'Donnell DE, Casaburi R, Vincken W, Puente-Maestu L, Swales J, Lawrence D, Kramer B, Group Is (2011). Effect of indacaterol on exercise endurance and lung hyperinflation in COPD. Respir Med.

[31] Maltais F, Kirsten AM, Hamilton A, De Sousa D, Voss F, Decramer M. Evaluation of the effects of olodaterol on exercise endurance in patients with chronic obstructive pulmonary disease: results from two 6-week crossover studies. Respir Res. 2016;17(77) 10.1186/s12931-016-0389-5 PMC4936013 27383762

[32] O'Donnell DE, Fluge T, Gerken F, Hamilton A, Webb K, Aguilaniu B, Make B, Magnussen H (2004). Effects of tiotropium on lung hyperinflation, dyspnoea and exercise tolerance in COPD. Eur Respir J.

[33] Casaburi R, Kukafka D, Cooper CB, Witek TJ, Jr., Kesten S: Improvement in exercise tolerance with the combination of tiotropium and pulmonary rehabilitation in patients with COPD**.** Chest 2005, 127**:**809–817. 10.1378/chest.127.3.809 15764761

[34] Maltais F, Hamilton A, Marciniuk D, Hernandez P, Sciurba FC, Richter K, Kesten S, O'Donnell D (2005). Improvements in symptom-limited exercise performance over 8 h with once-daily tiotropium in patients with COPD. Chest.

[35] Travers J, Laveneziana P, Webb KA, Kesten S, O'Donnell DE (2007). Effect of tiotropium bromide on the cardiovascular response to exercise in COPD. Respir Med.

[36] Maltais F, Celli B, Casaburi R, Porszasz J, Jarreta D, Seoane B, Caracta C (2011). Aclidinium bromide improves exercise endurance and lung hyperinflation in patients with moderate to severe COPD. Respir Med.

[37] Beeh KM, Singh D, Di Scala L, Drollmann A (2012). Once-daily NVA237 improves exercise tolerance from the first dose in patients with COPD: the GLOW3 trial. Int J Chron Obstruct Pulmon Dis.

[38] Cooper CB, Celli BR, Jardim JR, Wise RA, Legg D, Guo J, Kesten S (2013). Treadmill endurance during 2-year treatment with tiotropium in patients with COPD: a randomized trial. Chest.

[39] Beeh KM, Watz H, Puente-Maestu L, de Teresa L, Jarreta D, Caracta C, Garcia Gil E, Magnussen H. Aclidinium improves exercise endurance, dyspnea, lung hyperinflation, and physical activity in patients with COPD: a randomized, placebo-controlled, crossover trial. BMC Pulm Med. 2014;14(209) 10.1186/1471-2466-14-209 PMC4364572 25539654

[40] Casaburi R, Maltais F, Porszasz J, Albers F, Deng Q, Iqbal A, Paden HA, O'Donnell DE, Investigators (2014). Effects of tiotropium on hyperinflation and treadmill exercise tolerance in mild to moderate chronic obstructive pulmonary disease. Ann Am Thorac Soc.

[41] Beeh KM, Korn S, Beier J, Jadayel D, Henley M, D’Andrea P, Banerji D (2014). Effect of QVA149 on lung volumes and exercise tolerance in COPD patients: the BRIGHT study. Respir Med.

[42] O'Donnell DE, Sciurba F, Celli B, Mahler DA, Webb KA, Kalberg CJ, Knobil K (2006). Effect of fluticasone propionate/salmeterol on lung hyperinflation and exercise endurance in COPD. Chest.

[43] Worth H, Forster K, Eriksson G, Nihlen U, Peterson S, Magnussen H (2010). Budesonide added to formoterol contributes to improved exercise tolerance in patients with COPD. Respir Med.

[44] Guenette JA, Webb KA, O'Donnell DE (2013). Effect of fluticasone/salmeterol combination on dyspnea and respiratory mechanics in mild-to-moderate COPD. Respir Med.

[45] Calverley PM, Anderson JA, Celli B, Ferguson GT, Jenkins C, Jones PW, Yates JC, Vestbo J (2007). Salmeterol and fluticasone propionate and survival in chronic obstructive pulmonary disease. N Engl J Med.

[46] Bateman ED, Ferguson GT, Barnes N, Gallagher N, Green Y, Henley M, Banerji D (2013). Dual bronchodilation with QVA149 versus single bronchodilator therapy: the SHINE study. Eur Respir J.

[47] Vogelmeier C, Zhong N, Humphries MJ, Mezzi K, Fogel R, Bader G, Patalano F, Banerji D (2016). Indacaterol/glycopyrronium in symptomatic patients with COPD (GOLD B and GOLD D) versus salmeterol/fluticasone: ILLUMINATE/LANTERN pooled analysis. Int J Chron Obstruct Pulmon Dis.

[48] Calzetta L, Ora J, Cavalli F, Rogliani P, O'Donnell DE, Cazzola M (2017). Impact of LABA/LAMA combination on exercise endurance and lung hyperinflation in COPD: a pair-wise and network meta-analysis. Respir Med.

[49] Puente-Maestu L, Palange P, Casaburi R, Laveneziana P, Maltais F, Neder JA, O'Donnell DE, Onorati P, Porszasz J, Rabinovich R (2016). Use of exercise testing in the evaluation of interventional efficacy: an official ERS statement. Eur Respir J.

[50] Laviolette L, Bourbeau J, Bernard S, Lacasse Y, Pepin V, Breton MJ, Baltzan M, Rouleau M, Maltais F. Assessing the impact of pulmonary rehabilitation on functional status in COPD. Thorax. 2008(63):115–21. 10.1136/thx.2006.076844 17901158

[51] Puente-Maestu L, Villar F, de Miguel J, Stringer WW, Sanz P, Sanz ML, de Pedro JG, Martinez-Abad Y (2009). Clinical relevance of constant power exercise duration changes in COPD. Eur Respir J.

[52] Uronis HE, Ekstrom MP, Currow DC, DC MC, Samsa GP, Abernethy AP (2015). Oxygen for relief of dyspnoea in people with chronic obstructive pulmonary disease who would not qualify for home oxygen: a systematic review and meta-analysis. Thorax.

[53] Zhang X, Waterman LA, Ward J, Baird JC, Mahler DA (2010). Advantages of endurance treadmill walking compared with cycling to assess bronchodilator therapy. Chest.

