# Correction to: Long-acting bronchodilators improve exercise capacity in COPD patients: a systematic review and meta-analysis

**DOI:** 10.1186/s12931-018-0764-5

**Published:** 2018-04-24

**Authors:** Fabiano Di Marco, Giovanni Sotgiu, Pierachille Santus, Denis E. O’Donnell, Kai-Michael Beeh, Simone Dore, Maria Adelaide Roggi, Lisa Giuliani, Francesco Blasi, Stefano Centanni

**Affiliations:** 1 Respiratory Unit, Ospedale San Paolo, Department of Health Science, Università degli Studi di Milano, Via A. di Rudinì, 8-20142 Milan, Italy; 2 Clinical Epidemiology and Medical Statistics Unit, Department of Biomedical Sciences, University of Sassari, Sassari, Italy; 3 Department of Biomedical And Clinical Sciences (DIBIC), University of Milan, Milan, Italy; 4 Respiratory Unit, “Luigi Sacco” University Hospital; ASST Fatebenefratelli-Sacco, Milan, Italy; 5 Division of Respiratory and Critical Care Medicine, Respiratory Investigation Unit, Queen’s University and Kingston General Hospital, Kingston, ON Canada; 6 Insaf Respiratory Research Institute, Wiesbaden, Germany; 7 Cardiothoracic and Vascular Department, University of Pisa, Pisa, Italy; 8 Department of Pathophysiology and Transplantation, University of Milan, Milan, Italy; 9 Internal Medicine Department, Respiratory Unit and Cystic Fibrosis Adult Center, Fondazione IRCCS Ca’ Granda Ospedale Maggiore Policlinic, Milan, Italy

## Correction

In the original publication [1] is an incorrect sentence in the abstract under Conclusions. The modified text is indicated in **bold**. The correct version can be found in this Erratum:

Incorrect version:

Conclusions: Long-acting bronchodilators improve exercise capacity in COPD. The main effect of long-acting bronchodilators seems to be a **decrease** of basal IC rather than a modification of dynamic hyperinflation during exercise. The efficacy in terms of endurance time seems higher in studies which enrolled patients with hyperinflation, with a similar efficacy on walking or cycling.

Correct version

Conclusions: Long-acting bronchodilators improve exercise capacity in COPD. The main effect of long-acting bronchodilators seems to be an **increase** of basal IC rather than a modification of dynamic hyperinflation during exercise. The efficacy in terms of endurance time seems higher in studies which enrolled patients with hyperinflation, with a similar efficacy on walking or cycling.
