# Supplementary material for: Long-acting bronchodilators improve exercise capacity in COPD patients: a systematic review and meta-analysis
Source: Respir Res. 2018 Jan 24;19:18. doi: 10.1186/s12931-018-0721-3 (PMC5784692; doi:10.1186/s12931-018-0721-3)
Supplement: Additional file 1: Figure S1. — Funnel plots for ET, isotime IC and isotime dyspnea. Figure S2A Forest and Funnel plots of ET, isotime IC and isotime dyspnea in the 11 studies which included only COPD patients with functional residual capacity (FRC) > 120%. Figure S2B Forest and Funnel plots of ET, isotime IC and isotime dyspnea in the 11 studies which did not require an increase of functional residual capacity (FRC) as inclusion criterion (i.e. unselected COPD patients). Figure S3 Forest and Funnel plots of ET of the 5 studies (A) which used walking, and 17 studies (B) which used cycling as exercise methodology to assess the efficacy of long-acting bronchodilators. Table S1 Cochrane collaboration tool for assessing risk of bias. (DOCX 545 kb) [file 12931_2018_721_MOESM1_ESM.docx]

Long-acting bronchodilators improve exercise capacity in COPD patients: a systematic review and meta-analysis

Fabiano **Di Marco**^1^, Giovanni **Sotgiu**^2^, Pierachille **Santus**^3^, Denis E. **O’Donnell**^4^, Kai-Michael **Beeh**^5^, Simone **Dore**^2^, Maria Adelaide **Roggi**^6^, Lisa **Giuliani**^1^, Francesco **Blasi**^7^, and Stefano **Centanni**^1^

**Additional file**

**Figure S1.** Funnel plots for ET, isotime IC and isotime dyspnea (all studies).

**Endurance time**

**
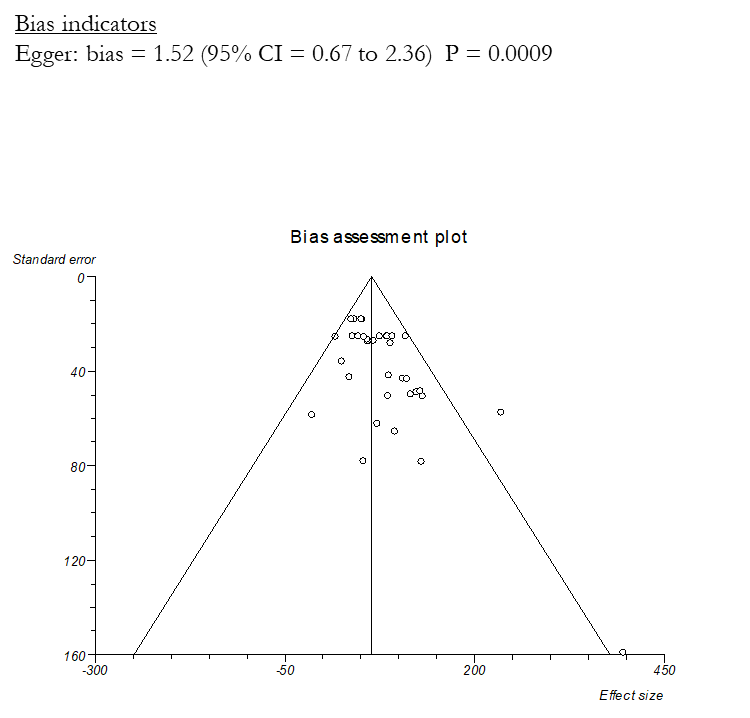
**

**Isotime inspiratory capacity**

**
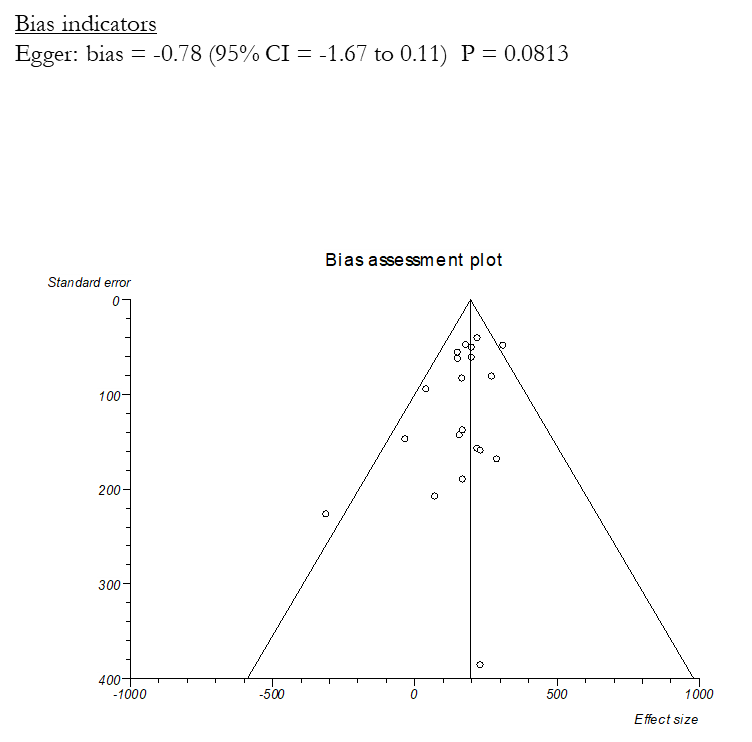
**

**Isotime dyspnea BORG**

**
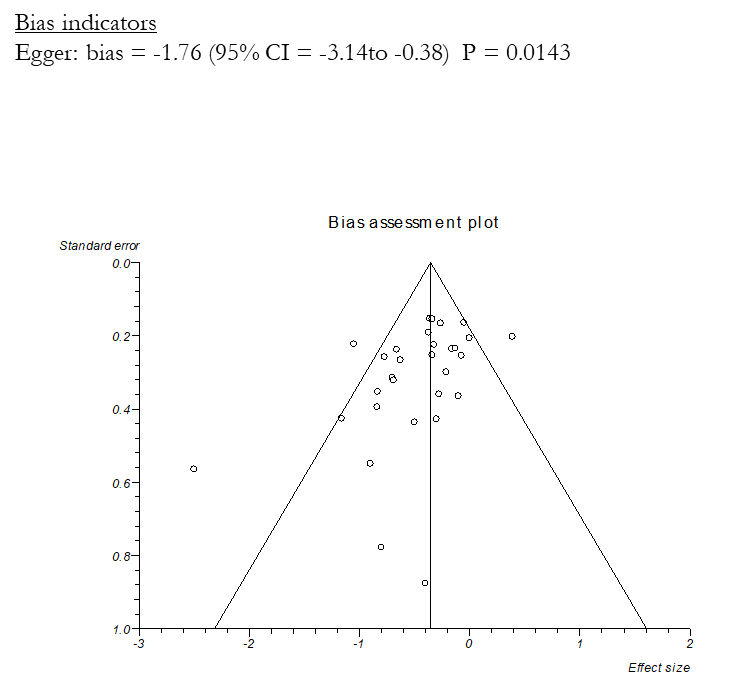
**

**Figure S2 A.** Forest and Funnel plots of ET, isotime IC and isotime dyspnea in the 11 studies which included only COPD patients with functional residual capacity (FRC) > 120%.

**Endurance time**

**
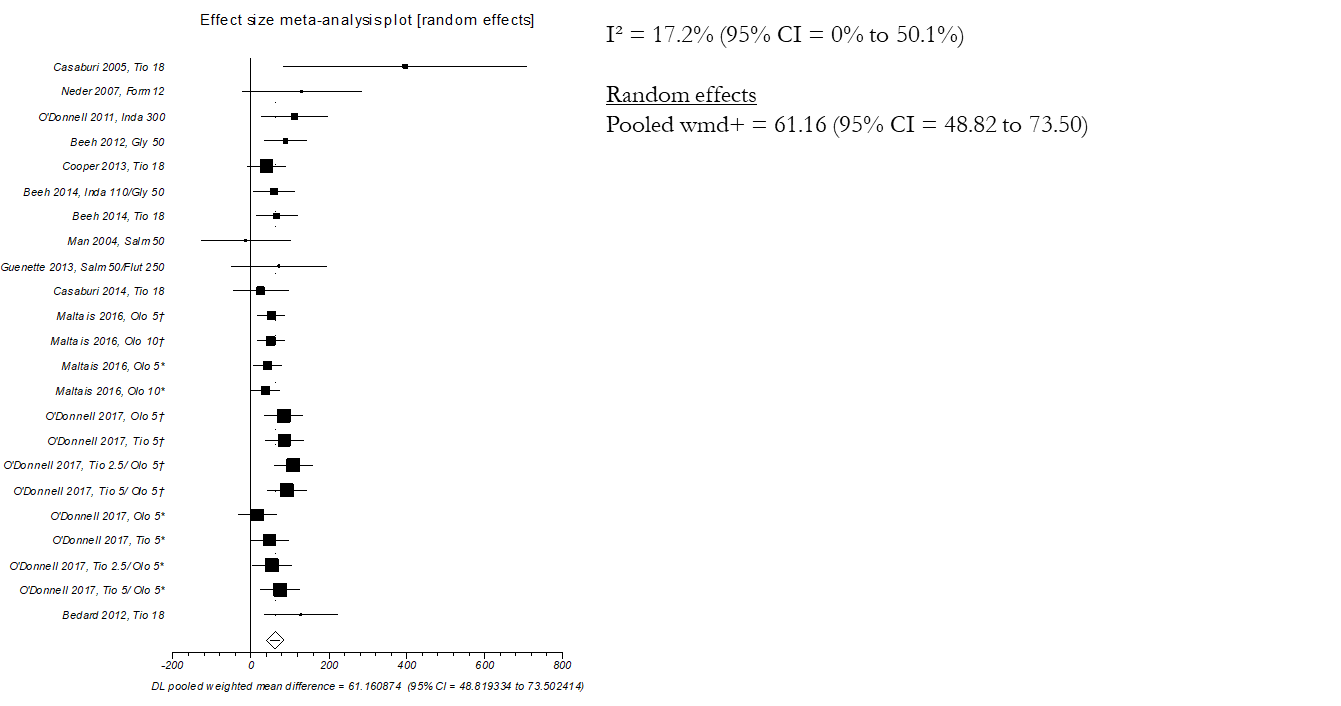
**

**
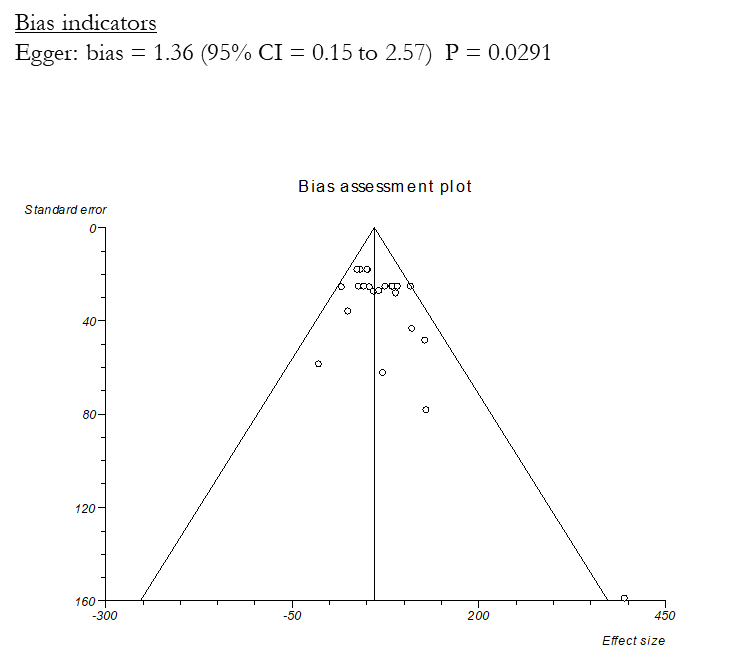
**

**Isotime inspiratory capacity**

**
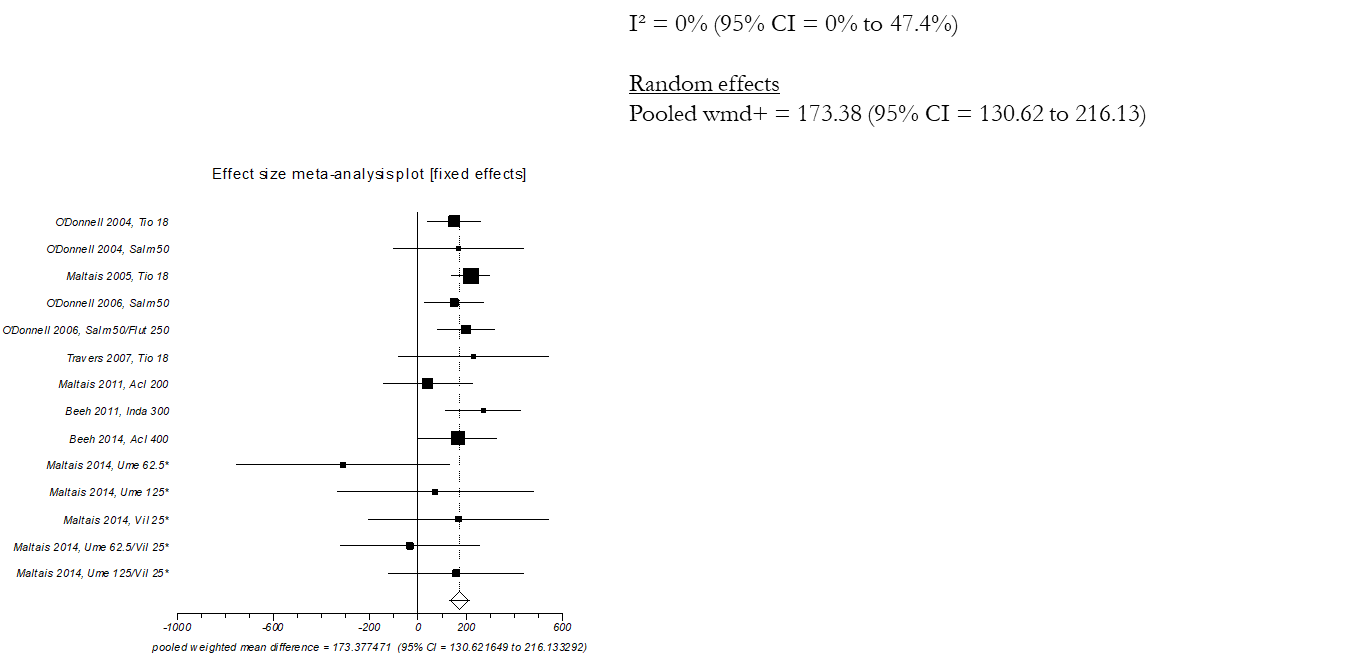
**

**
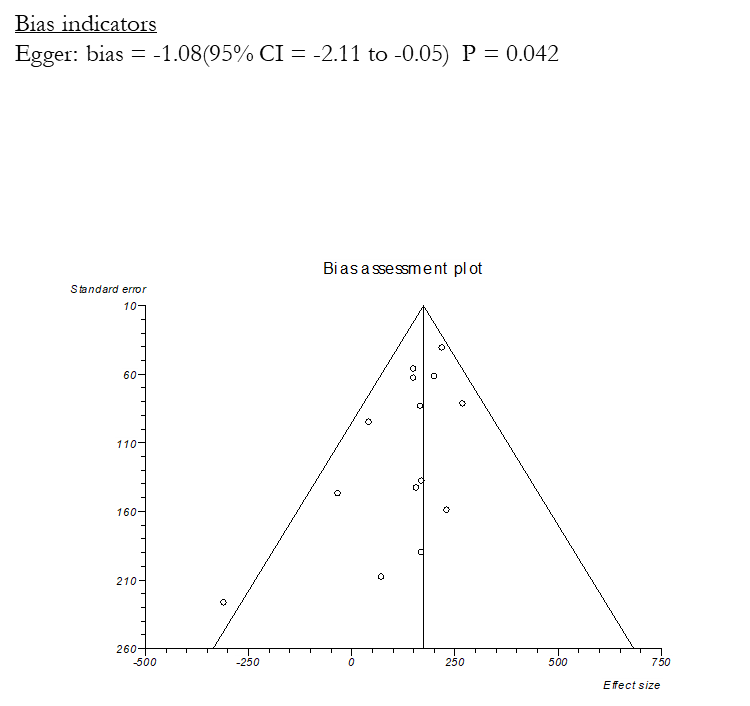
**

**Isotime dyspnea BORG**

**
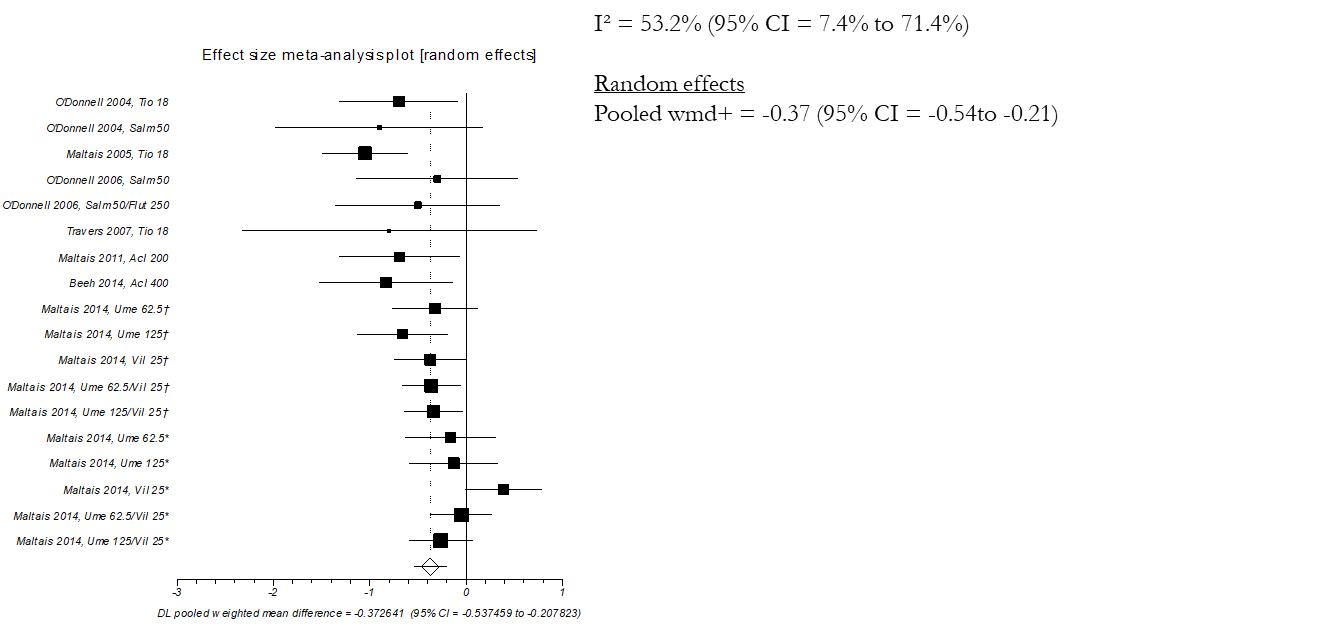
**

**
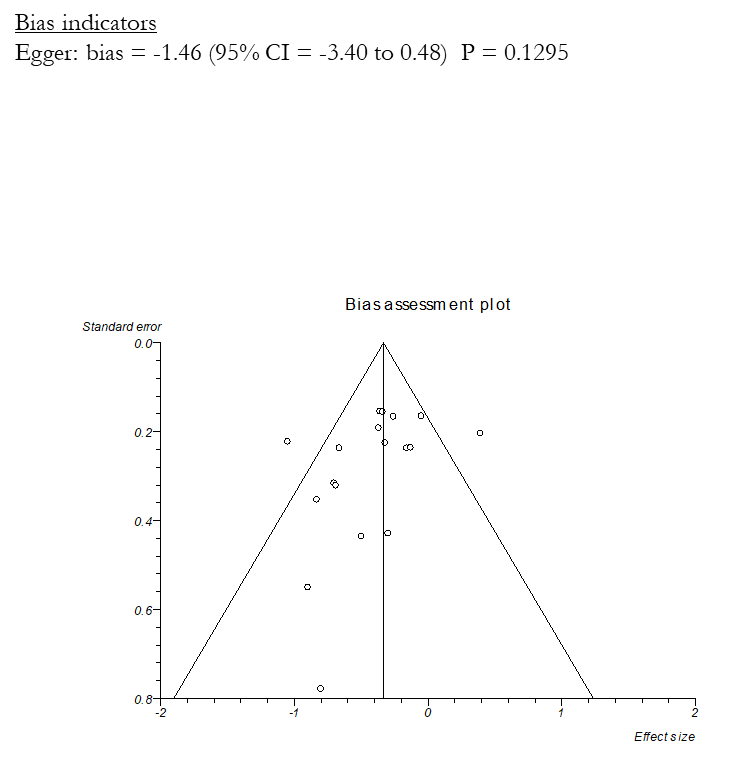
**

**Figure S2 B.** Forest and Funnel plots of ET, isotime IC and isotime dyspnea in the studies which did not require an increase of functional residual capacity (FRC) as inclusion criterion.

**Endurance time**

**
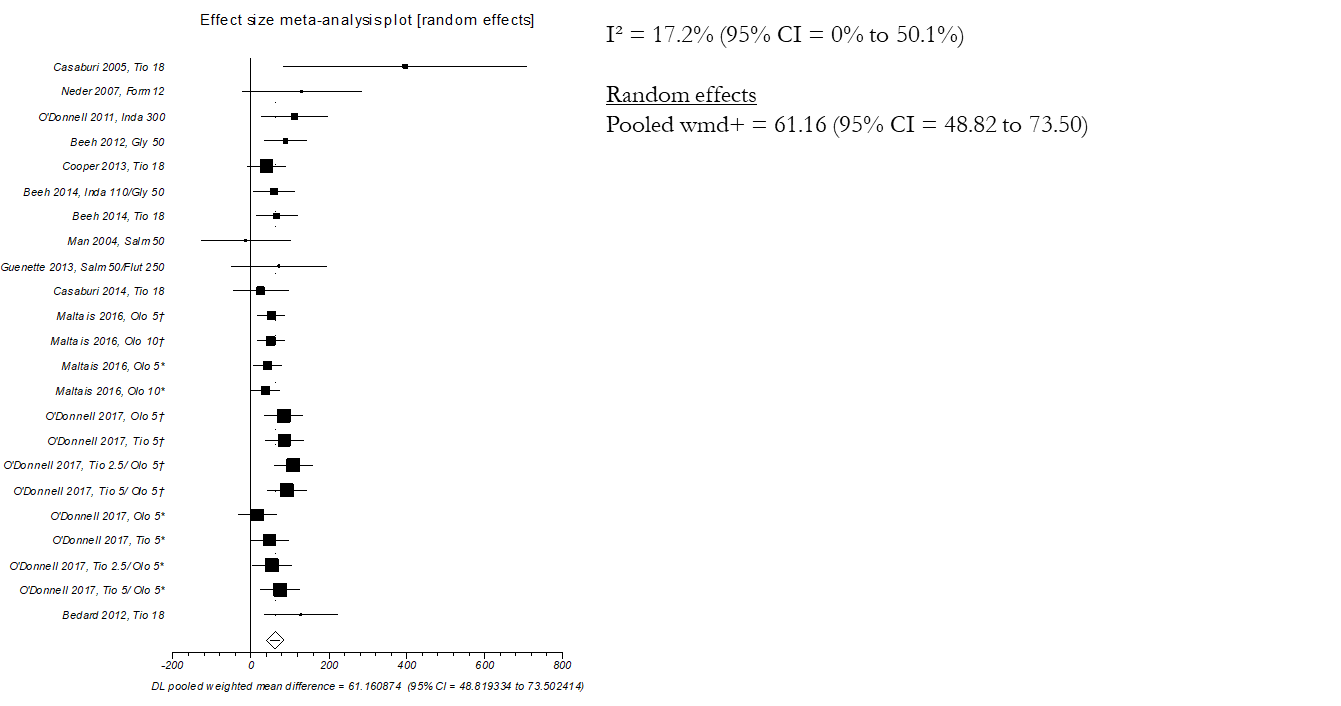
**

**
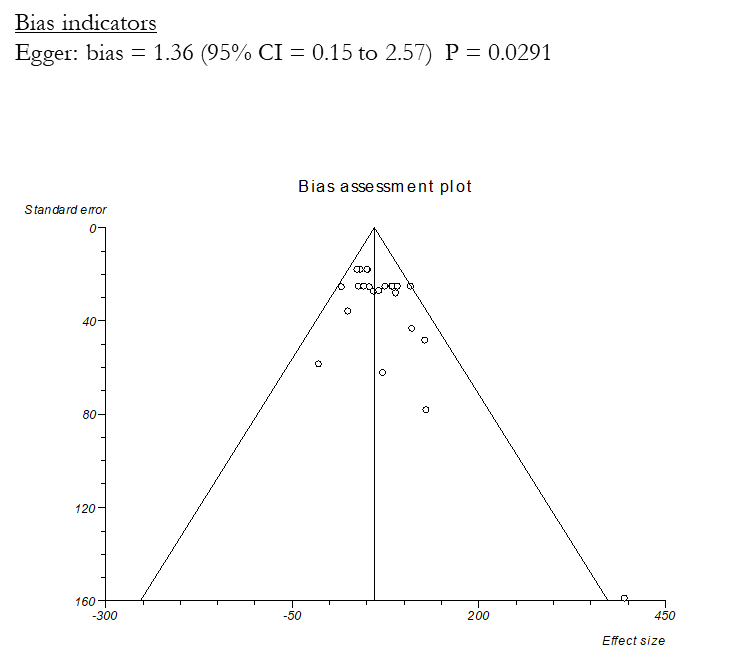
**

**Isotime inspiratory capacity**

**
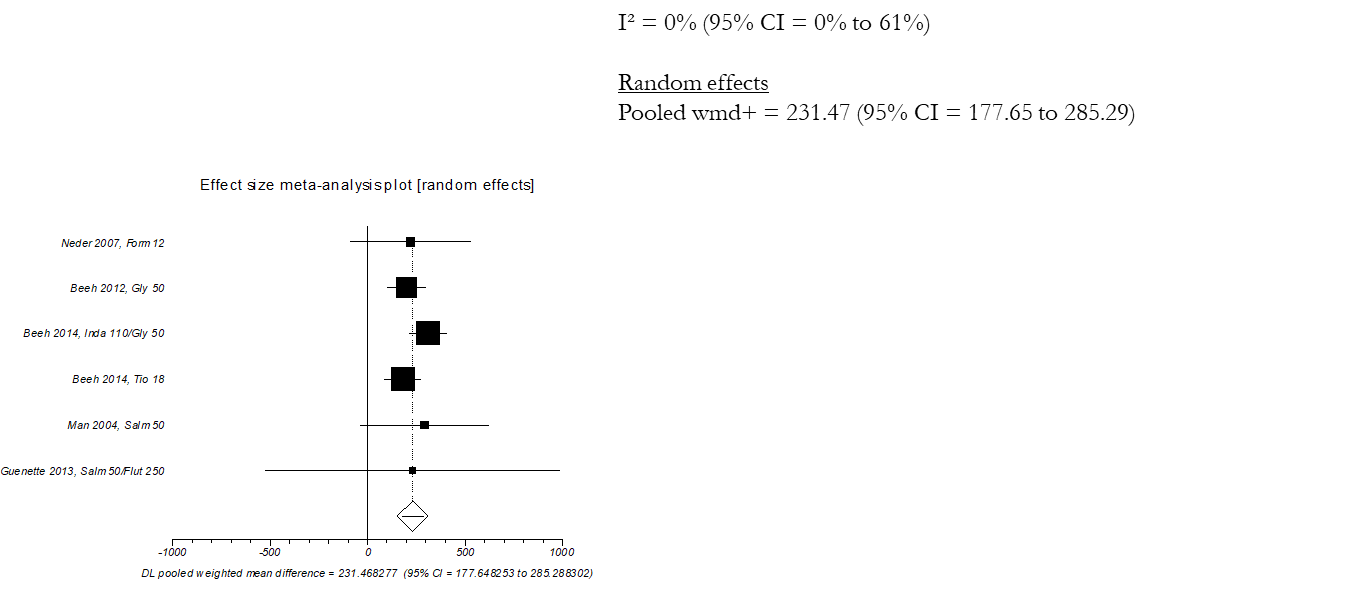
**

**
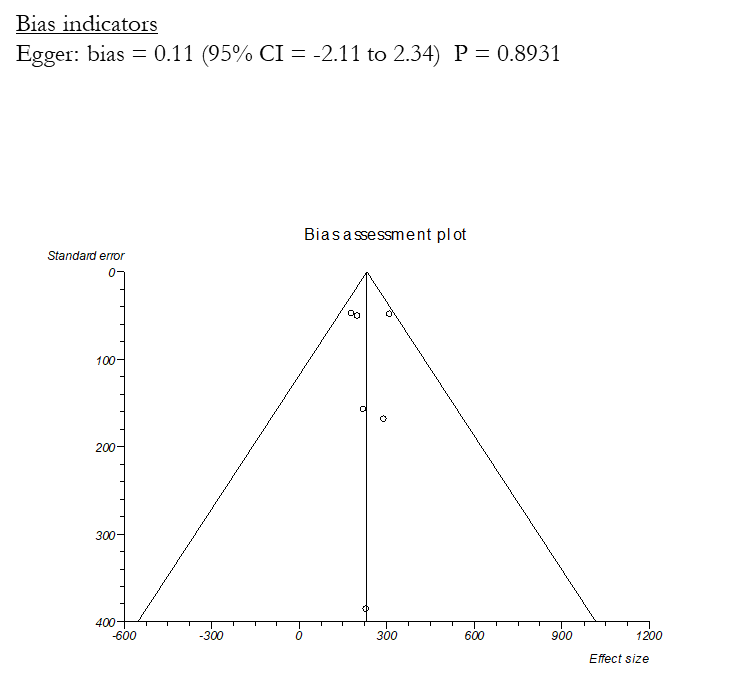
**

**Isotime dyspnea BORG**

**
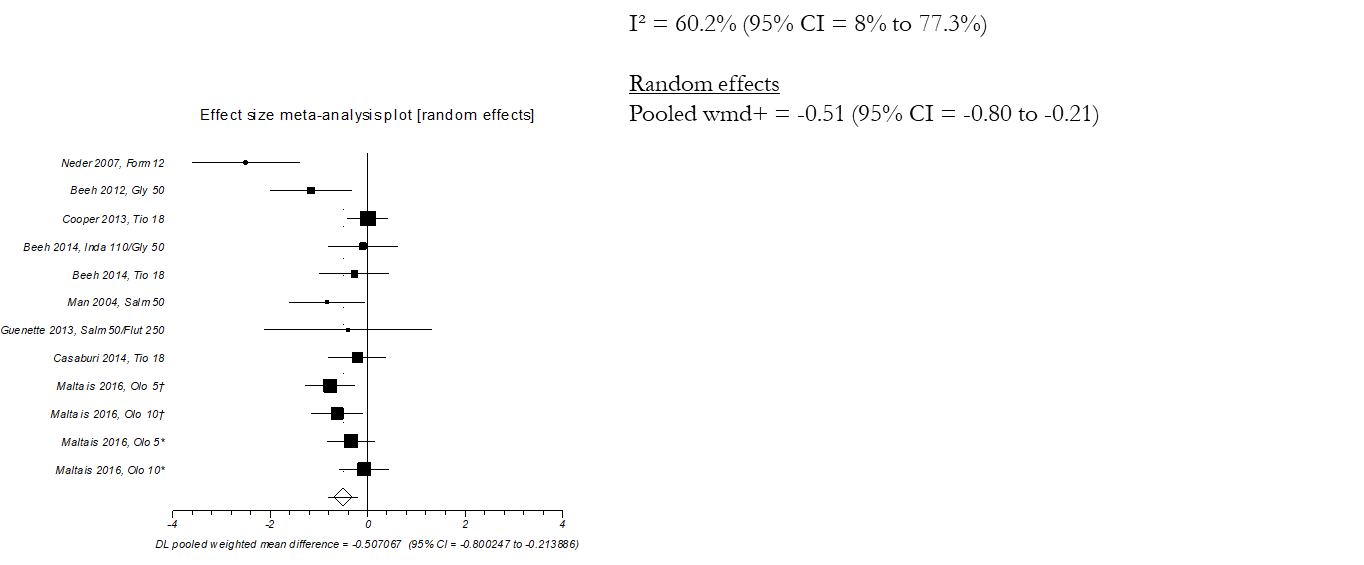
**

**
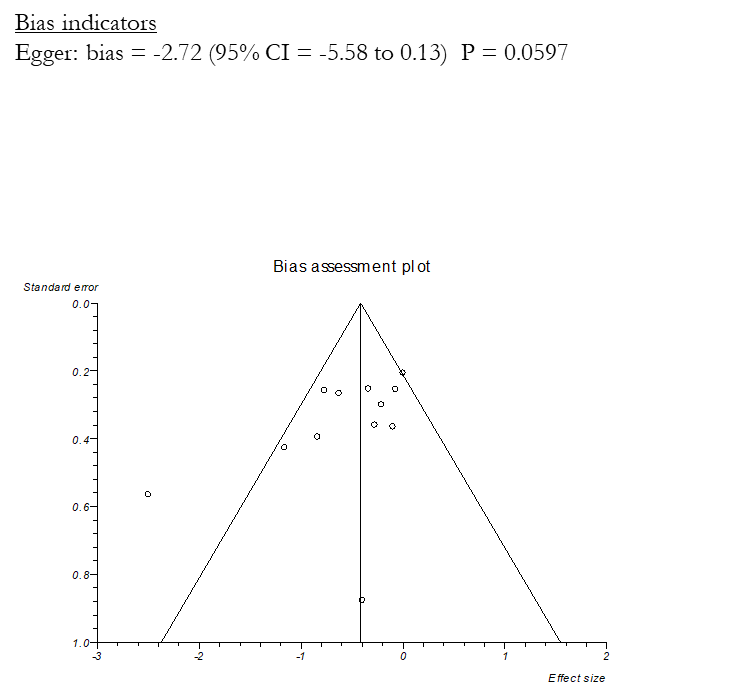
**

**Figure S3.** Forest and Funnel plots of ET of the 5 studies (A) which used walking and 17 studies (B) which used cycling as exercise methodology to assess the efficacy of long-acting bronchodilators.

**A)**

**
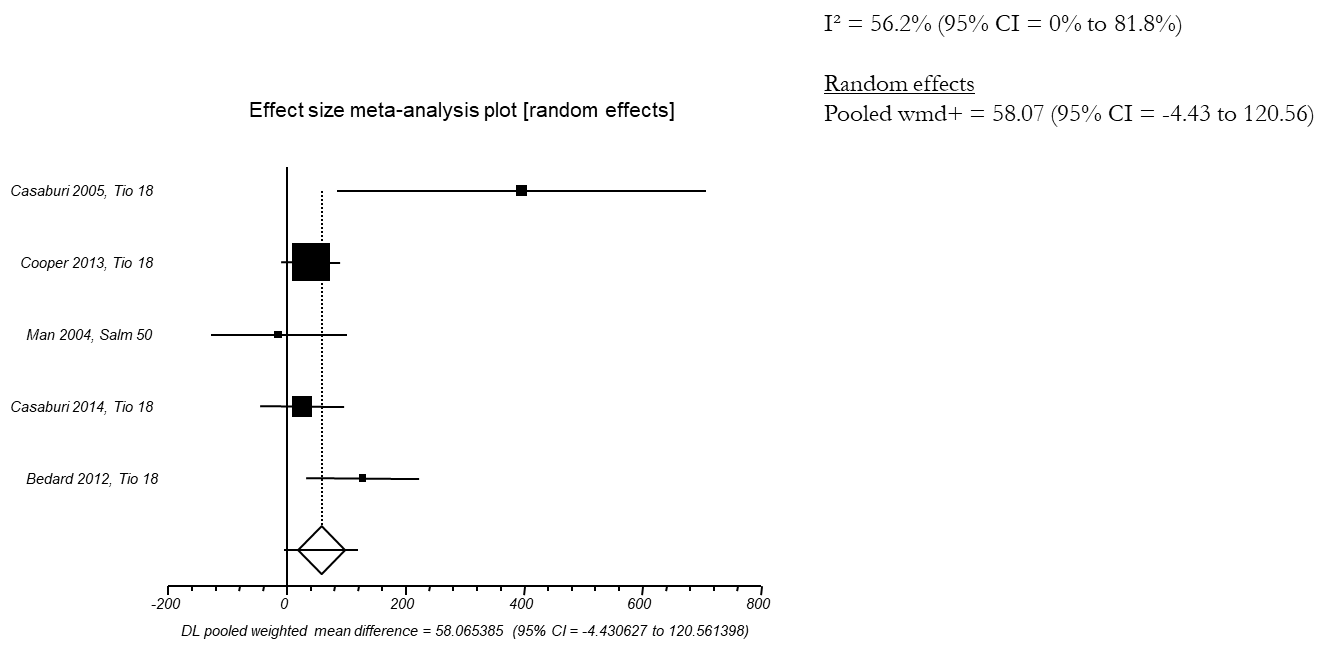
**

**
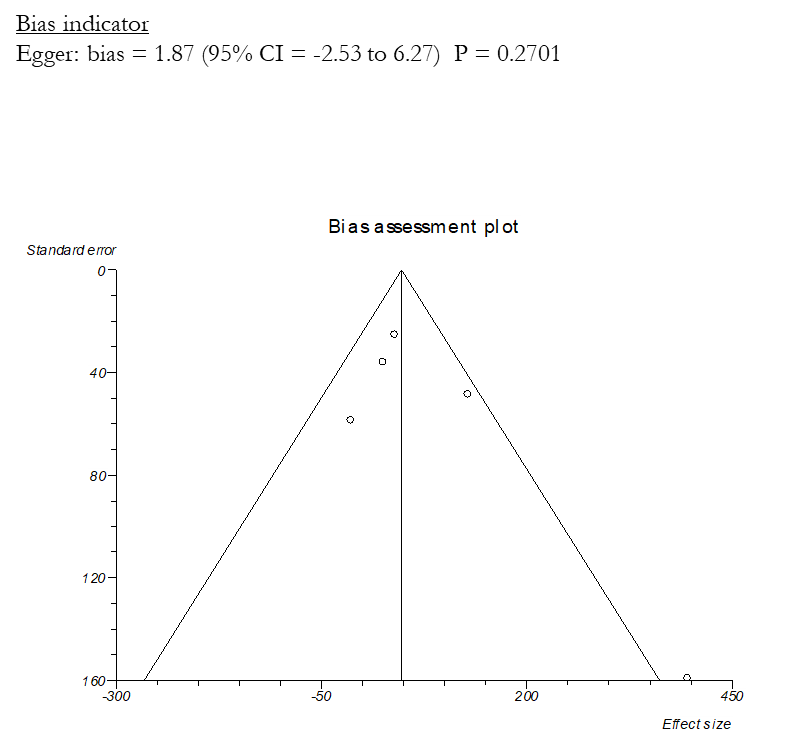
**

**B)**

**
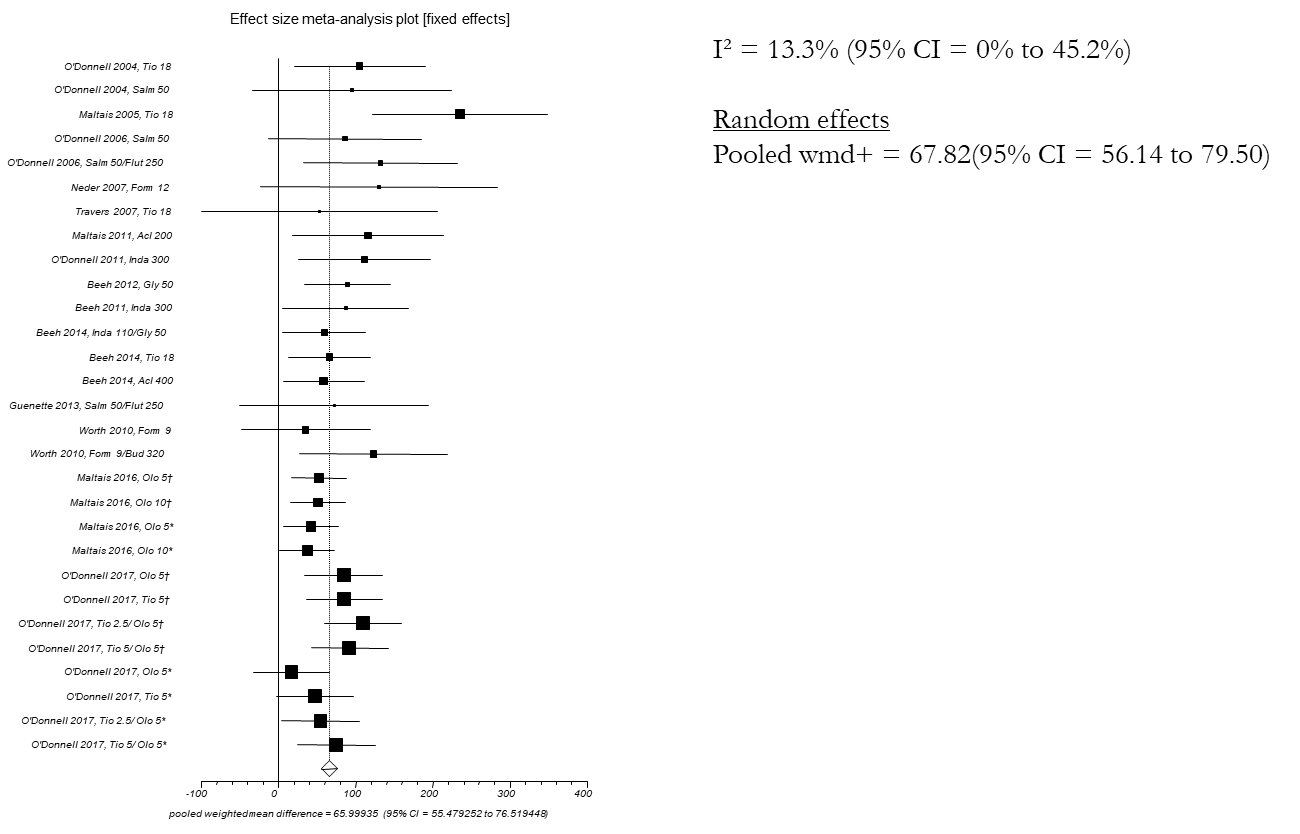
**

**
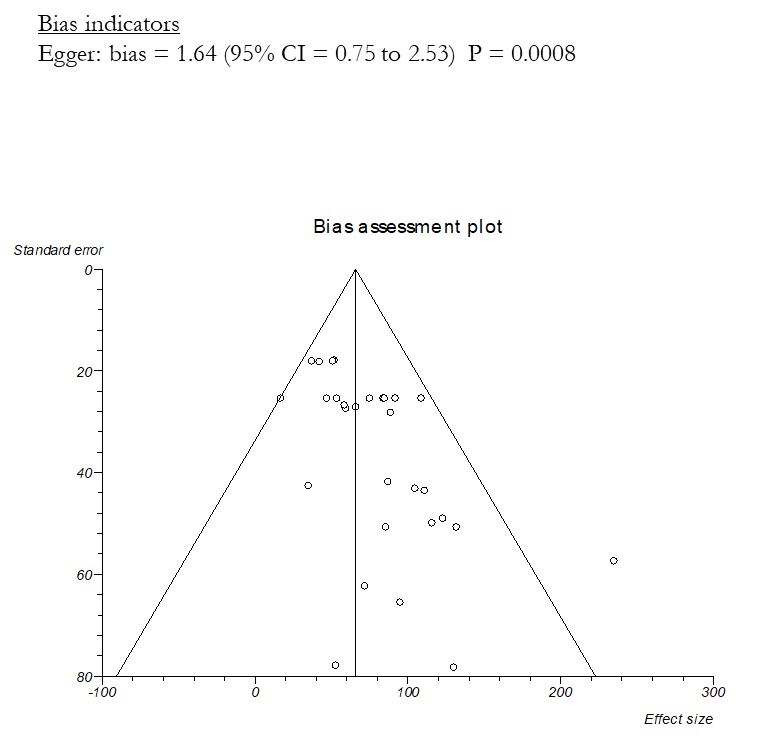
**

**Table S1.** Cochrane collaboration tool for assessing risk of bias.


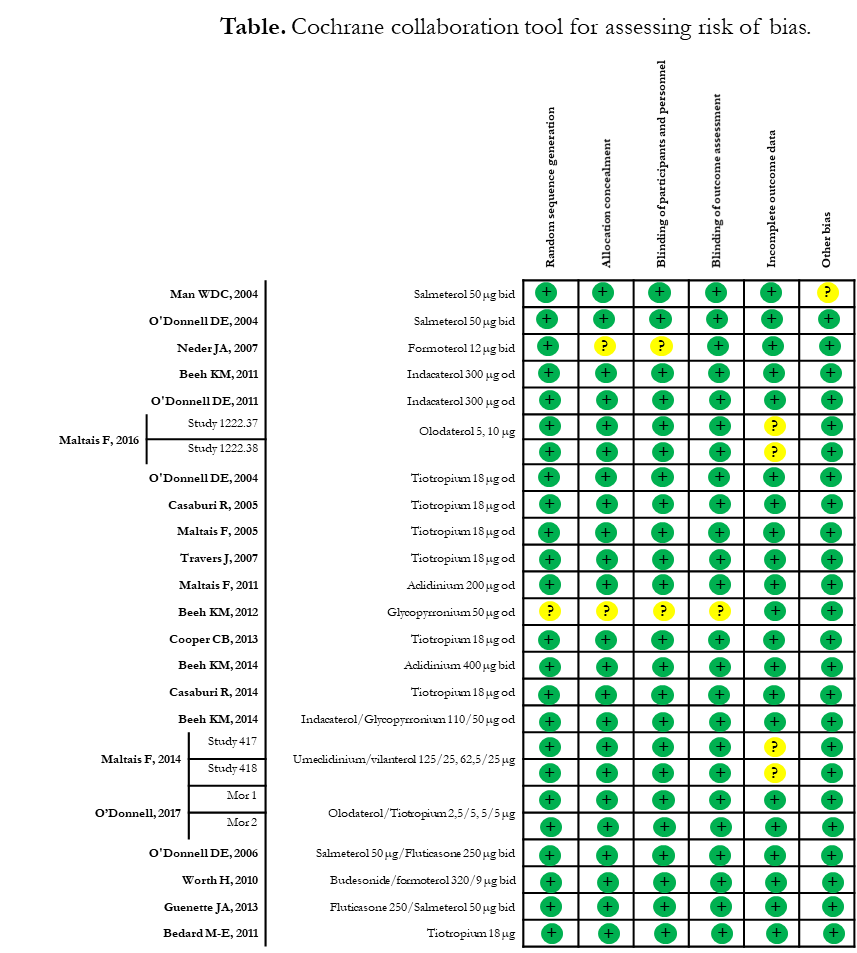


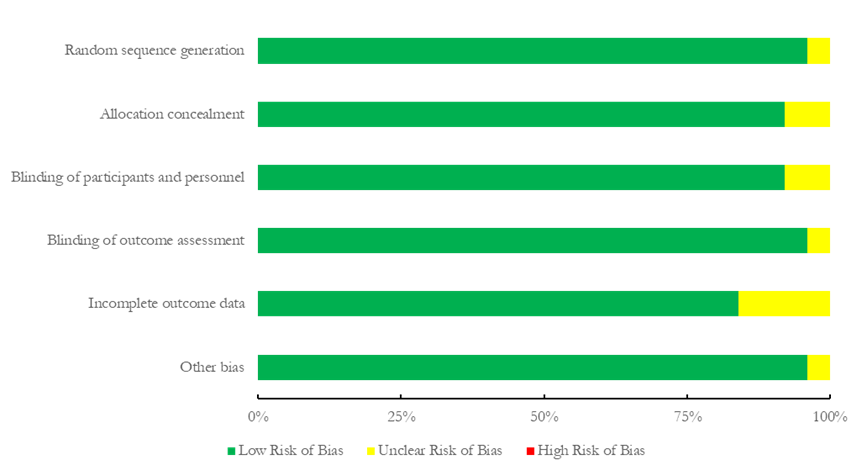


**Table S2**. List of and reason why studies on long-acting bronchodilators have been excluded from the analysis.

| **Study** | **Reason for exclusion** |
| --- | --- |
| Canto ND, et al. Addition of tiotropium to formoterol improves inspiratory muscle strength after exercise in COPD. Respir Med 2012; 106: 1404–1412. | No placebo arm |
| Tzani P, et al.[Effects of beclomethasone/formoterol fixed combination on lung hyperinflation and dyspnea in COPD patients.](https://www.ncbi.nlm.nih.gov/pubmed/22069361) Int J Chron Obstruct Pulmon Dis. 2011;6:503-9. | No placebo arm |
| [Berton DC](https://www.ncbi.nlm.nih.gov/pubmed/?term=Berton%20DC%5BAuthor%5D&cauthor=true&cauthor_uid=20580216), et al. Effects of tiotropium and formoterol on dynamic hyperinflation and exercise endurance in COPD. [Respir Med.](https://www.ncbi.nlm.nih.gov/pubmed/20580216) 2010 Sep;104(9):1288-96. | No placebo arm |
| [Magnussen H](https://www.ncbi.nlm.nih.gov/pubmed/?term=Magnussen%20H%5BAuthor%5D&cauthor=true&cauthor_uid=22749044), et al. Effect of combination treatment on lung volumes and exercise endurance time in COPD. [Respir Med.](https://www.ncbi.nlm.nih.gov/pubmed/?term=magnussen+2012+copd+endurance+time) 2012 Oct;106(10):1413-20. | No placebo arm |
| Yoshimura K, et al. Effects of tiotropium on sympathetic activation during exercise in stable chronic obstructive pulmonary disease patients. Int J Chron Obstruct Pulmon Dis 2012; 7: 109–117. | No placebo arm |
| [Brouillard C](https://www.ncbi.nlm.nih.gov/pubmed/?term=Brouillard%20C%5BAuthor%5D&cauthor=true&cauthor_uid=18057052), et al. Endurance shuttle walking test: responsiveness to salmeterol in COPD. [Eur Respir J.](https://www.ncbi.nlm.nih.gov/pubmed/18057052) 2008 Mar;31(3):579-84. | Duration < 1 week |
| [van der Vaart H](https://www.ncbi.nlm.nih.gov/pubmed/?term=van%20der%20Vaart%20H%5BAuthor%5D&cauthor=true&cauthor_uid=21660300), et al. Bronchodilation improves endurance but not muscular efficiency in chronic obstructive pulmonary disease. [Int J Chron Obstruct Pulmon Dis.](https://www.ncbi.nlm.nih.gov/pubmed/?term=van+der+vaart+grevink+postma) 2011;6:229-35. | Data expressed only as difference vs. placebo and not high intensity protocol |
| [Watz H](https://www.ncbi.nlm.nih.gov/pubmed/?term=Watz%20H%5BAuthor%5D&cauthor=true&cauthor_uid=28883722), et al. ACTIVATE: the effect of aclidinium/formoterol on hyperinflation, exercise capacity, and physical activity in patients with COPD. [Int J Chron Obstruct Pulmon Dis.](https://www.ncbi.nlm.nih.gov/pubmed/?term=watz+activate+copd+aclidinium) 2017 Aug 24;12:2545-2558. | Data expressed only as difference vs. placebo |
| [Zhang X](https://www.ncbi.nlm.nih.gov/pubmed/?term=Zhang%20X%5BAuthor%5D&cauthor=true&cauthor_uid=20040610), et al. Advantages of endurance treadmill walking compared with cycling to assess bronchodilator therapy. [Chest.](https://www.ncbi.nlm.nih.gov/pubmed/?term=zhang+chest+2010+endurance+time+copd) 2010 Jun;137(6):1354-61. | Data expressed only as difference vs. placebo |
